# Operando Surface Spectroscopy and Microscopy during Catalytic Reactions: From Clusters via Nanoparticles to Meso‐Scale Aggregates

**DOI:** 10.1002/smll.202004289

**Published:** 2021-03-10

**Authors:** Günther Rupprechter

**Affiliations:** ^1^ Institute of Materials Chemistry Technische Universität Wien Getreidemarkt 9/BC/01 Vienna 1060 Austria

**Keywords:** clusters, heterogeneous catalysis, interfaces, nanoparticles, operando, surface science

## Abstract

Operando characterization of working catalysts, requiring per definitionem the simultaneous measurement of catalytic performance, is crucial to identify the relevant catalyst structure, composition and adsorbed species. Frequently applied operando techniques are discussed, including X‐ray absorption spectroscopy, near ambient pressure X‐ray photoelectron spectroscopy and infrared spectroscopy. In contrast to these area‐averaging spectroscopies, operando surface microscopy by photoemission electron microscopy delivers spatially‐resolved data, directly visualizing catalyst heterogeneity. For thorough interpretation, the experimental results should be complemented by density functional theory. The operando approach enables to identify changes of cluster/nanoparticle structure and composition during ongoing catalytic reactions and reveal how molecules interact with surfaces and interfaces. The case studies cover the length‐scales from clusters via nanoparticles to meso‐scale aggregates, and demonstrate the benefits of specific operando methods. Restructuring, ligand/atom mobility, and surface composition alterations during the reaction may have pronounced effects on activity and selectivity. The nanoscale metal/oxide interface steers catalytic performance via a long ranging effect. Combining operando spectroscopy with switching gas feeds or concentration‐modulation provides further mechanistic insights. The obtained fundamental understanding is a prerequisite for improving catalytic performance and for rational design.

## Graphical abstract

Operando surface spectroscopy and microscopy enable molecular level insight into ongoing catalytic reactions. Employing X‐ray, photoelectron and infrared based spectroscopy, the active catalyst state can be determined, including reaction‐induced changes. Cluster and nanoparticle restructuring, atom/ligand mobility and surface composition alterations have pronounced effects on catalytic performance. Surface microscopy directly reveals how reactions initiate and spatially evolve.

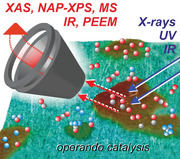

## 1 Introduction

Heterogeneous catalysis is a traditional field of nanotechnology, as in many cases catalysts are composed of initially termed “small metal particles” supported by meso‐/nanoscale oxides.^[^ ^1^, ^2^, ^3^, ^4^, ^5^, ^6^, ^7^, ^8^, ^9^ ^]^ Because catalytic reactions proceed on the surface of the materials, a high surface‐to‐volume ratio is beneficial. This can be described as metal dispersion (number of metal surface atoms relative to the total number of metal atoms in the catalyst), whereas the support is characterized by the specific surface area (m^2^ g^−1^). For hemispherical metal particles of ≈1.5, 2, 5, and 10 nm diameter, the dispersion is about 0.8, 0.6, 0.3, and 0.1, respectively, illustrating why the nanoscale is required not to waste precious metal inside the particles. The ultimate dispersion is, of course, provided by single metal atoms and this concept is followed in “single atom catalysis” or “single site catalysis”.^[^ ^10^, ^11^, ^12^, ^13^, ^14^, ^15^, ^16^, ^17^ ^]^ However, the adsorption and reaction steps of a catalytic reaction do typically not occur on isolated single atoms, but also involve neighboring sites, for example, of the oxide support^[^ ^18^ ^]^ or of a metal matrix (for bimetallic nanoparticles, active metal atoms may be embedded in an inert or less‐active metal matrix^[^ ^11^, ^19^, ^20^, ^21^, ^22^, ^23^ ^]^). The so‐called site isolation of active atoms may prevent C—C bond cleavage (coking), which requires at least two neighboring active metals. In any case, the stability of the single atoms is the key challenge, as atoms may diffuse and sinter. Furthermore, for example, Pt, Pd, Cu, or Au atoms are mobilized by CO,^[^ ^24^, ^25^, ^26^, ^27^, ^28^ ^]^ forming CO‐M_1_ units that may eventually sinter into larger clusters.

**Figure 1:**
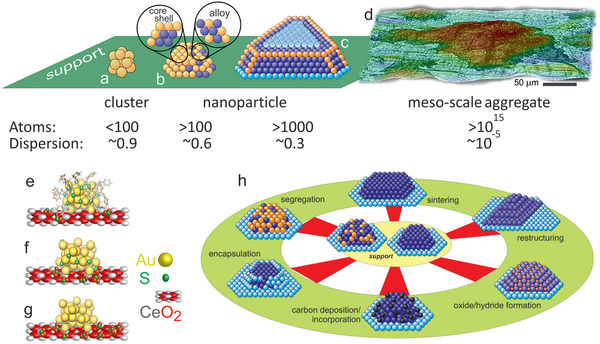
Bridging length‐scales of supported catalysts from clusters via nanoparticles to meso‐sized aggregates. The models illustrate the orders of magnitude: a) cluster of 11 atoms (with all but one on the surface), b) ≈2 nm nanoparticle with >100 atoms (with about 60% on the surface), c) ≈5 nm nanoparticle with >1000 atoms (with about 30% on the surface) and d) ≈100 µm aggregate with >10^15^ atoms (with about 10^−3^% on the surface). e–g) display ligand removal and restructuring of supported Au_38_ clusters upon oxidative treatments. In (h), some possible structural and compositional changes of catalysts are illustrated. e–g) Adapted with permission.^[^ ^99^ ^]^ Copyright 2018, Wiley‐VCH. h) Adapted with permission.^[^ ^100^ ^]^ Copyright 2008, IOP Publishing.

Herein, we define metal clusters as entities with less than ≈100 atoms, characterizing the so‐called “non‐scalable” regime (**Figure** **1**a).^[^ ^29^ ^]^ Their atomic and electronic structure is significantly different from that of bulk metals, for example, with respect to atom positions (icosahedral vs face centered cubic *fcc*), distances (lattice contraction or expansion), and band structure (semiconductor vs metal). Clearly, these properties are size‐dependent (“each atom counts”^[^ ^30^, ^31^ ^]^), with pronounced impact on adsorption and reaction properties.^[^ ^5^, ^32^ ^]^ Accordingly, nanoparticles with more than ≈100 atoms are in the “scalable‐regime” and start to have or exhibit bulk atomic and electronic structure.^[^ ^6^, ^32^, ^33^, ^34^ ^]^ Nevertheless, the relative contributions of corner, edge, step, terrace and phase boundary sites on the surface of a nanoparticle (Figure 1b,c) are still size‐dependent.^[^ ^35^, ^36^ ^]^ Meso‐scale (“black”) powders of metals are clearly bulk‐like and have very low dispersion (Figure 1d), but the µm‐sized aggregates still consist of adjoining nanocrystals with structured surfaces, comparable to that of true nanoparticles.

It is well‐know that the type of support used to carry the atoms/clusters/nanoparticles has a pronounced effect on catalytic performance.^[^ ^5^, ^37^, ^38^, ^39^, ^40^, ^41^, ^42^, ^43^ ^]^ For inert supports the effect may be indirect by affecting the size, shape and (interfacial) oxidation state of the metal, whereas for active supports some reaction steps may additionally occur on the support or at the metal/support interface (bifunctional catalysis). For meso‐scale aggregates one would not expect any influence of the support, as the contribution of perimeter sites is minute, but this must be revised, a shown below.

Oxide supported atoms, clusters, and nanoparticles can be characterized by various methods. Transmission or scanning electron microscopy (TEM/SEM) have been used to determine particle size distributions (histograms), but smallest particles were often undetectable, especially for oxides with strong contrast.^[^ ^44^, ^45^, ^46^, ^47^, ^48^ ^]^ In this case, chemisorption of probe molecules (H_2_, CO) provided at least an average dispersion, from which a mean particle diameter can be calculated (based on a couple of assumptions).^[^ ^49^ ^]^ Progress in microscopy nowadays enables to detect even single atoms by aberration‐corrected high‐resolution transmission electron microscopes.^[^ ^16^, ^44^, ^50^ ^]^ Further characterization of phases, coordination numbers (CN) and interatomic distances (*R*) can be performed by X‐ray absorption spectroscopy (XAS)^[^ ^51^, ^52^, ^53^, ^54^, ^55^ ^]^ and X‐ray diffraction.^[^ ^56^, ^57^, ^58^, ^59^, ^60^ ^]^ X‐ray photoelectron spectroscopy (XPS) yields information on catalyst (surface) composition and adsorbed species.^[^ ^61^, ^62^, ^63^, ^64^, ^65^, ^66^ ^]^ The same holds for infrared spectroscopy (Fourier transform (FT) IR),^[^ ^20^, ^24^, ^60^, ^67^, ^68^, ^69^, ^70^, ^71^, ^72^, ^73^, ^74^, ^75^, ^76^ ^]^ but the its focus is clearly on vibrations of adsorbed reactant/intermediate/spectator/product molecules. For brevity, XRD^[^ ^56^, ^57^, ^58^, ^59^, ^60^ ^]^ and Raman spectroscopy^[^ ^77^, ^78^, ^79^, ^80^, ^81^ ^]^ are not discussed in here, despite their importance.

When such nanoparticles/clusters were employed in catalysis, the traditional approach was to characterize them before and after the reaction. This revealed that the catalyst structure and composition often change during the reaction and examples of such changes are presented in Section 2. But how much can one learn about the “catalytic symphony” by visiting the concert hall before and after the performance? Consequently, catalysts should or even must be examined during the ongoing reaction, with simultaneous product analysis, and the combination of the two defines the operando approach, as explained in detail below. The application of operando surface spectroscopy (e.g., XAS, XPS, IR, with simultaneous MS analysis) directly correlates a specific catalyst state with its resulting activity/selectivity. This enables one to monitor the evolution of the as‐prepared catalyst to an “active state” under reaction conditions, which may help to identify reactive phases, reaction mechanisms, and strategies how to improve activity, selectivity, and lifetime. Based on the operando results, the synthesis, processing and operation routes may be refined or revised, and may even lead to a rational catalyst design.^[^ ^82^ ^]^

However, there is another limitation when studies of working catalysts are carried out by area‐averaging spectroscopic methods. Structurally different locations of a catalyst will have different catalytic properties, but this local information is inaccessible by averaging techniques. Clearly, advances were made by local measurements via, for example, micro‐spectroscopy.^[^ ^51^, ^83^, ^84^, ^85^, ^86^, ^87^ ^]^ If many sample positions are analyzed and combined, elemental composition or other “maps” can be constructed, but this is usually too slow to catch fast spatially‐correlated processes.

In contrast to ensemble‐averaging methods, microscopies provide spatially‐resolved information on different catalyst positions in every image frame, with spatial and time resolution depending on the microscope, including in situ (environmental)/operando HRTEM or SEM,^[^ ^50^, ^88^, ^89^, ^90^, ^91^, ^92^, ^93^ ^]^ and photoemission electron microscopy (PEEM).^[^ ^86^, ^94^, ^95^, ^96^, ^97^, ^98^ ^]^ HRTEM characterizes catalysts structure/composition/morphology, whereas PEEM mainly yields information on the spatial distribution of adsorbates and on reaction processes (reaction fronts, kinetic transitions, oscillations, etc.). Based on the parallel‐imaging principle of HRTEM and PEEM, all positions within the field of view are imaged simultaneously (in contrast to scanning methods) and real‐time videos of surface processes can be obtained, creating a “molecular movie of catalysts at work”.^[^ ^51^, ^85^ ^]^

In the following, the operando approach and selected methods will be highlighted and subsequently illustrated by several case studies bridging the size range from clusters via nanoparticles to meso‐scale aggregates. Operando surface spectroscopy and surface microscopy, applied during catalytic reactions with simultaneous product analysis, are essential to directly reveal cluster/nanoparticle restructuring (structure collapse, atom mobility, intracluster segregation) and how molecules interact and react at surfaces and interfaces (including steady‐state and time‐resolved measurements). Ex situ studies could apparently not provide this kind of information (or just to a limited extent). The operando studies also illustrate how cluster/nanoparticle synthesis, activation, processing, and metal/support interactions can be utilized to tune catalytic performance.

## 2 The Operando Approach

The traditional approach of catalyst development and evaluation includes at least these steps: i) synthesis of nanostructured materials (routes/processing, loading, promotion, etc.), ii) basic characterization before and after activating pretreatments (i.e., before reaction, with respect to nanoparticle size, shape, structure, composition, specific surface area, porosity, adsorption/desorption properties, etc.), iii) evaluation of catalytic performance (activity, selectivity, reaction orders, activation energies, stability/lifetime, etc.), iv) post‐reaction characterization to determine reaction‐induced changes, v) cycling and re‐activation, and vi) optimization/modification. For all steps there are many variables and combinations of parameters, and their selection is typically guided by experience and knowledge, but this still has the flavor of trial‐and‐error. This complexity was a motivation of high‐throughput screening, which can be commercially successful and has begun impacting in academia,^[^ ^101^, ^102^, ^103^ ^]^ but requires large infrastructure investments.

Returning to curiosity‐driven fundamental catalysis research, one should note that the characterization of an as‐prepared and activated (pretreated) catalyst is clearly important, but not truly relevant if the catalyst changes under reaction conditions. The same holds for post‐reaction analysis, unless the active catalyst state can be quenched and is unchanged until characterization is performed. Pre‐ and post‐reaction characterization is thus only indirectly related to the active state present during reaction, if at all. Nevertheless, pre‐ and post‐reaction characterization have demonstrated that catalysts can evolve in many ways (Figure 1e‐g,h):

i) Cluster and nanoparticle restructuring: reactive conditions may induce atom and ligand migration, as well as intraparticle restructuring (Figure 1e‐g), as discussed for supported Au clusters in detail below. Upon reaction, particle size (sintering, redispersion), shape (e.g., polyhedral to rounded and vice versa), and the metal/support interface (encapsulation, spreading) may be modified (Figure 1h). Accordingly, these effects are long‐standing topics in catalysis.^[^ ^39^, ^40^, ^46^, ^104^, ^105^ ^]^
ii) Alterations in surface composition: for monometallics, surface oxides, carbide‐like phases, or hydrides may be formed or vanish during catalytic reactions.^[^ ^106^, ^107^, ^108^, ^109^, ^110^, ^111^, ^112^, ^113^, ^114^ ^]^ For bimetallics, segregation of one constituent may be induced by strong‐bonding to a reactant or a (by‐)product.^[^ ^115^, ^116^ ^]^ As shown below, CH*_x_* species formed from adsorbed CH_4_ on CuNi alloys induce Ni surface segregation and turn the intended surface composition “upside‐down”. Similarly, during selective methanol steam reforming (MSR) on PdZn or PdGa intermetallics, the by‐product CO drives more Pd to the surface, thereby partly decomposing the selective phase.^[^ ^108^, ^117^, ^118^, ^119^ ^]^

Most of these effects have originally been detected by contrasting pre‐ and post‐reaction characterization. However, one can never be sure whether structures/compositions have only been formed or vanished after reaction, during cool‐down, and/or switching from reactive to inert gas. For example, the occurrence and stability of surface oxides or hydrides critically depends on the oxygen and hydrogen pressure, respectively. If the catalyst activity/selectivity changes during time‐on‐stream, which is common, post‐reaction characterization would even be required for many run times and is even less meaningful.

Consequently, over the last decades there were intensive efforts to perform catalyst characterization during the ongoing catalytic reaction. At this point, terminology needs to be discussed, differentiating in situ and operando. Researchers have used the term in situ for spectroscopy, diffraction or microscopy carried out under reaction conditions. However, despite correct pressure/temperature conditions, catalytic performance was often not recorded in parallel. The latter may originate from complications of appropriately combining catalyst characterization with reactant/product gas phase analysis (especially at synchrotron beamlines), apart from the fact that standard in situ cells are often not being compatible with suitable (flow) reactor geometry.^[^ ^119^ ^]^ However, without gas phase/product analysis one cannot directly relate structure/composition/adsorbates to catalytic performance and, in the worst case, the catalyst performs differently during spectroscopy/diffraction/microscopy and in kinetic reactor tests.

To add to the confusion, the term in situ has also been used for studies when pretreatment and catalysis were performed inside a dedicated cell, without exposing the sample to air in‐between, but characterization was in fact performed after and not during each process. For example, the acquisition of XAS spectra may require to cool down the catalyst or to evacuate the spectroscopy cell (but without opening it in between). This is different from ex situ, when several different batches of nominally the same catalyst (as‐prepared, pretreated, post‐reaction, aged, deactivated, re‐activated, etc.) are separately measured one after the other. It was suggested to use the term “studies of functioning catalysts”, but this still did not imply simultaneous recording of catalytic performance.

**Figure 2:**
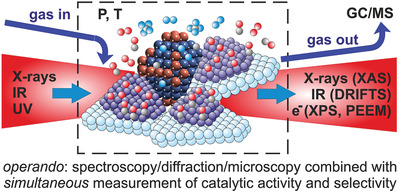
Key concept of the operando approach to heterogeneous catalysis. Using dedicated cells (dashed line), spectroscopy, microscopy or diffraction is performed on the catalyst under working conditions, while simultaneously monitoring catalytic performance, for example, by GC/MS analysis of reactants and products. Methods discussed below serve as examples.

Such considerations made a group of researchers coin a new term for studies combining catalysts characterization (spectroscopy/microscopy/diffraction) during ongoing reaction with simultaneous reactant/product analysis (**Figure** **2**): operando.^[^ ^120^, ^121^, ^122^, ^123^, ^124^, ^125^, ^126^ ^]^ This approach guarantees that the characterization data can be directly correlated with the catalytic performance (although one can still discuss about cell geometries, pressures etc.).^[^ ^119^ ^]^ Operando was originally intended to streamline terminology in catalysis research, but meanwhile the term became so popular that it is often used simply as a synonym of in situ, and even for non‐catalytic studies monitoring just one parameter (and in the worst case even wrongfully termed “in operando”).

## 3 Selected Operando Methods

In the following, selected operando characterization methods that are capable to examine catalysts under working conditions are highlighted. First, area‐averaging spectroscopy methods are discussed, before turning to spatially‐resolved surface microscopy. Each subsection covers the basics and strengths/limitations of the methods, including a few examples demonstrating why operando measurements are crucial to characterize active phases. Cell/sample geometries and dedicated technical implications are also presented.

### 3.1 X‐Ray Absorption Spectroscopy (XAS: XANES and EXAFS)

As X‐rays do not suffer from gas phase absorption and easily penetrate quartz or sapphire capillary reactors, as well as aluminum or Kapton windows of reactor cells, X‐ray absorption spectroscopy (XAS)^[^ ^127^ ^]^ is a powerful technique for probing the local geometric and electronic structure of catalysts in a wide parameter space (even to >1000 K and >100 bar). Catalysts can be evaluated during the various steps of synthesis and activation, but also during ongoing reaction. Prime examples of operando applications encompass reforming, hydrodesulfurization (HDS), hydrogenation, selective oxidation, and automotive catalysis, covering a wide range of materials (e.g., zeolites, sulfides, oxides, supported transition and noble metal clusters/nanoparticles).^[^ ^51^, ^52^, ^53^, ^54^, ^55^, ^85^, ^126^, ^127^, ^128^, ^129^, ^130^, ^131^ ^]^

In XAS, the X‐ray energy is tuned over an absorption edge and the photoelectron (wave) created at an atom scatters at neighboring atom(s), with the interference modulating the absorption cross‐section above the excitation threshold. While XANES (X‐ray Absorption Near‐Edge Structure; near‐edge region up to ≈100 eV post‐edge) provides information on phases, atomic/electronic structure and oxidation states, EXAFS (Extended X‐ray Absorption Fine Structure; ≈100–800 eV post‐edge) indicates exact bond lengths and coordination numbers (as a limitation, adsorbed molecules cannot be detected, though). XANES is also called NEXAFS (Near‐edge X‐ray Absorption Fine Structure), but this term will not be used herein.

**Figure 3:**
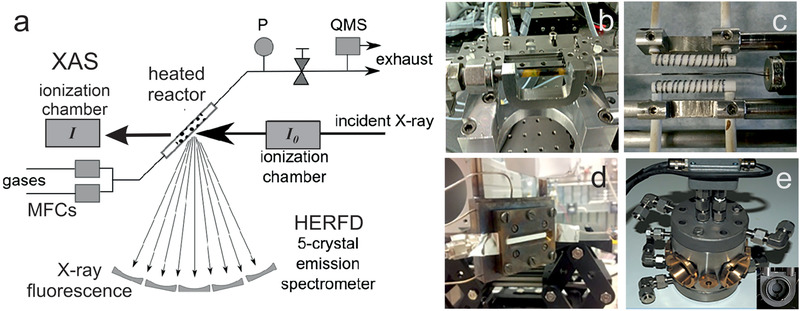
a) Illustration of operando X‐ray absorption spectroscopy (XAS) in transmission and fluorescence mode.^[^ ^139^ ^]^ Various cells are shown: b) quartz tube (Swiss Light Source), c) quartz capillary (TU Wien^[^ ^131b^ ^]^), d) transmission cell (MAX‐LAB II, l811), and e) fluorescence cell (ALBA, CLAESS), with the inset showing the pellet holder.

**Figure** **3**a shows a typical setup of a capillary reactor. Absorption of the incident X‐ray beam (*I*_0_) can be monitored by the attenuation of the transmitted photon beam (*I*), or via fluorescence as the core holes decay. Recent developments include fast spectral acquisition (quick‐EXAFS), spatial resolution (e.g., along the catalyst bed) and higher energy resolution (high energy resolution fluorescence detection; HERFD).^[^ ^51^, ^52^, ^53^, ^54^, ^55^, ^85^, ^132^, ^133^, ^134^, ^135^ ^]^ Figure 3b–d show various sample stages, with the catalyst powders contained in a tube, capillary and a void (between two Al windows), respectively. Figure 3e shows an operando XANES cell (with Kapton windows), with the inset displaying the holder for a pressed catalyst pellet.

Data analysis of near‐edge structures it typically performed by linear correlation fits based on reference compounds, whereas EXAFS analysis requires more elaborate fitting and modelling (scattering theory).^[^ ^136^, ^137^ ^]^ More details on XAS can be found in the literature.^[^ ^51^, ^52^, ^53^, ^54^, ^55^, ^85^, ^133^, ^134^, ^135^, ^136^, ^137^, ^138^ ^]^

### 3.2 Near Ambient Pressure X‐Ray Photoelectron Spectroscopy

In XPS, the catalyst surface is illuminated by an X‐ray beam and the kinetic energy of the created photoelectrons, from which their binding energy can be deduced, delivers information on catalyst elemental composition and valence band structure, but also on adsorbed species and the surrounding gas phase.^[^ ^140^, ^141^, ^142^ ^]^ Due to its inherent surface‐sensitivity resulting from the small inelastic mean path of the photoelectrons, X‐ray photoelectron spectroscopy is very versatile surface analysis method. Using synchrotron radiation not only provides better energy resolution, but tuning the photon energy and thus the photoelectron kinetic energy enables depth‐profiling of the near‐surface region (important for, e.g., subsurface species, layered or core‐shell structures). Resonantly‐enhanced photoemission can strongly increase sensitivity. For catalysis, the near ambient (atmospheric) pressure mode (NAP‐XPS) is most useful, as it can be applied during ongoing reactions in the few mbar pressure range, enabling to monitor changes in surface composition (e.g., segregation, alloying, coking, adsorbates). Early NAP‐XPS setups were lab‐based,^[^ ^143^, ^144^, ^145^, ^146^, ^147^ ^]^ but when combined with synchrotron radiation and differentially‐pumped electrostatic lenses round/after the year 2000, their number began to strongly increase.^[^ ^61^, ^62^, ^63^, ^64^, ^65^, ^66^, ^147^, ^148^ ^]^ Currently, operating pressures are limited to tens of mbar, but there are ongoing efforts to extend the pressure range. Further developments include micro‐spectroscopy and XPS‐microscopy^[^ ^83^, ^84^, ^86^ ^]^ and time‐resolved or even ultrafast spectroscopy.^[^ ^149^, ^150^ ^]^

NAP‐XPS is crucial to identify active phases that are only present under reaction conditions. For example, for “low temperature” (<800 °C) catalytic methane combustion on Pd/Al_2_O_3_, ex situ studies had suggested PdO (or PdO*_x_*) as active phase.^[^ ^151^, ^152^, ^153^ ^]^ However, the catalyst activates with time‐on‐stream and particle sintering and facetting were observed, along with hysteresis effects. Thus, the exact structure and composition of the active phase, including the possible contributions of (surface) oxides and metallic Pd, can hardly be deduced from ex situ characterization.

Thus, the formation of Pd oxides was intensively studied in situ,^[^ ^106^, ^112^, ^113^, ^154^, ^155^, ^156^, ^157^ ^]^ uncovering the transformation from a (2 × 2) chemisorbed oxygen adlayer via a 2D Pd_5_O_4_ surface oxide to PdO bulk oxide. The stability of the oxide phases sensitively depends on pressure and temperature, and a pronounced redox‐hysteresis was observed upon heating and cooling. For the activity maximum of CH_4_ combustion on Pd(111) at 350 °C, operando NAP‐XPS identified a novel surface phase consisting of PdO seeds on a Pd_5_O_4_ surface oxide, governed by a delicate balance of seed formation and reduction by methane.^[^ ^158^ ^]^ Below 350 °C, this active phase had not yet (fully) developed. Above 350 °C, PdO decomposes and the reaction proceeds on metallic Pd, but with its near‐surface region saturated by dissolved oxygen. Interestingly, upon cooling both the activity maximum and the PdO seeds/Pd_5_O_4_ phase were not observed.^[^ ^158^ ^]^ Similarly, a delicate balance of carbon formation (coking) and oxidation determines CO oxidation activity of Pt/ZrO_2_, as revealed by operando NAP‐XPS/MS (and sum frequency generation (SFG)).^[^ ^159^, ^160^ ^]^ Along similar lines, Pd‐carbide‐like phases (Pd*_x_* C*_y_*), with carbon remaining in the subsurface region and Pd acting as carbon “sponge”^[^ ^108^, ^109^, ^110^, ^114^ ^]^ were solely formed during selective hydrogenation reactions, with their abundance affecting activity. For all these examples, such complex temperature‐ and sample prehistory (hysteresis)‐dependent catalytic performance can only be observed and explained by operando studies.

**Figure 4:**
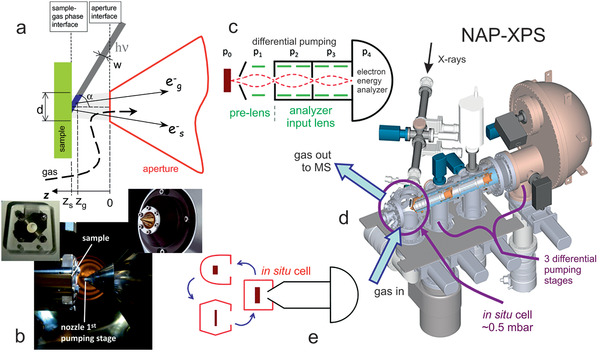
Illustration of operando near ambient pressure X‐ray photoelectron spectroscopy (NAP‐XPS):^[^ ^61^, ^62^ ^]^ a) measurement geometry and b) sample (holder)‐nozzle arrangement, (c) shows a differentially‐pumped (pre‐)lense system, d) overview of NAP‐XPS at HZB/BESSY II (ISISS), and e) interchangeable in situ cells, a) Adapted with permission.^[^ ^61^ ^]^ Copyright 2010, Elsevier. c, d) Adapted with permission.^[^ ^62^ ^]^ Copyright 2013, The Royal Society of Chemistry.

**Figure** **4** displays the key ingredients of a NAP‐XPS setup. As the photoelectrons can only travel a few millimeters at mbar gas pressure, the nozzle of the electron energy pre‐lens is very close to the sample (e.g., a pressed catalyst pellet mounted on a sapphire holder; Figure 4a,b). Strong differential pumping by various stages reduces the pressure by 10^9^ until the electrons reach the hemispherical electron energy analyzer (Figure 4c). Compared to the early lab‐based systems, the pre‐lens, and analyzer‐input lenses increase the acceptance angle and thus the number of collected photoelectrons, reducing the acquisition time. Figure 4d shows the complete setup, with the catalyst located in a cell, that also acts as flow reactor. To reduce reactor size, there may be a smaller cell around the sample (cell‐in‐cell design) and several different interchangeable cells may enable different applications (e.g., electrochemistry, tribology etc.; Figure 4e). For further details refer to other studies.^[^ ^61^, ^62^, ^63^, ^64^, ^65^ ^]^

### 3.3 Infrared Spectroscopy (FTIR, DRIFTS, ATR)

**Figure 5:**
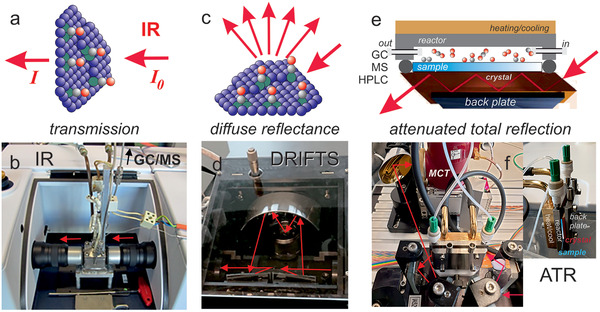
Illustration of operando infrared spectroscopy in a,b) transmission, c,d) diffuse reflectance, and e,f) attenuated total reflection mode. Cell design and IR beam paths are shown in the lower row (b,d,f).

Vibrational infrared (IR) spectroscopy of adsorbed species and the catalyst material itself is a versatile operando method,^[^ ^67^, ^68^, ^73^, ^74^, ^75^, ^85^, ^161^, ^162^, ^163^, ^164^, ^165^, ^166^ ^]^ based on the absorption of broadband IR radiation (*I*_0_) as measured by Fourier‐transform (FT) spectrometers. As IR is not surface‐specific, keeping the IR beam path in the gas phase short limits IR gas phase absorption (gas phase bands hold information on reactants and products, but may obscure surface species). Various catalytic IR cells for different measurement geometries (depending on the IR transmissivity of the samples) are commercially available, including transmission, diffuse reflectance and attenuated total reflection (FTIR, DRIFTS, and ATR, respectively), as schematically shown in **Figure** **5**. KBr or CaF_2_ windows and ZnSe or Ge ATR crystals are frequently used. For transmission and diffuse reflectance, catalyst powders are pressed to pellets or into small crucibles, respectively, for ATR the crystals are coated with thin catalyst films.

Once more, using IR in operando mode is crucial for revealing active species, phases and mechanisms. Operando FTIR spectra of CO oxidation on Co_3_O_4_ spinel revealed monodentate carbonates, but they turned out to be rather spectators, in light of their high thermal stability.^[^ ^59^ ^]^ During MSR on supported Pd_2_Ga nanoparticles (aiming for CO_2_ and H_2_ as products), part of the intermetallic surface decomposed to metallic Pd.^[^ ^60^, ^118^ ^]^ This is due to adsorbed CO formed as by‐product, that drives more Pd to the surface, which continuously reduces the CO_2_ selectivity of the catalyst. IR spectroscopy can also be favorably combined with concentration‐modulation (modulation‐excitation^[^ ^72^, ^167^, ^168^ ^]^), enabling to differentiate active (dynamic) and spectator (steady) species adsorbed on the catalyst (for details see Section 4.4 below).

For other in situ/operando vibrational spectroscopy of active catalyst materials we refer to the literature (e.g., Raman^[^ ^77^, ^78^, ^79^, ^80^, ^81^, ^85^, ^122^, ^123^, ^124^, ^125^, ^163^ ^]^ and SFG^[^ ^159^, ^160^, ^169^, ^170^, ^171^, ^172^ ^]^). The same ATR setup can also be used to study solid/liquid interfaces.^[^ ^173^, ^174^, ^175^ ^]^

### 3.4 Photoemission Electron Microscopy

With the exception of extended surfaces of metal or oxide single crystal model catalysts, the surface of a catalyst is hardly structurally homogeneous (and even single crystals exhibit some local defects, such as steps or kinks). Apparently, every area‐averaging technique cannot differentiate between local inhomogeneities, which is why one often aims for structurally homogenous catalyst samples. Applying surface microscopy solves this problem and even benefits from structurally diverse catalysts. Photoemission electron microscopy (PEEM) is maybe the most prominent example,^[^ ^176^ ^]^ especially well‐known from visualizing oscillations in CO oxidation on platinum.^[^ ^94^, ^95^, ^96^, ^177^ ^]^ In addition, PEEM was applied to examine catalytic H_2_ oxidation^[^ ^86^, ^178^, ^179^, ^180^ ^]^ and NO reduction.^[^ ^181^, ^182^ ^]^ The major benefit of operando surface microscopy is most obvious, as one can directly see where and how the (re)action takes place. As discussed below, the initiation and propagation of catalytic reactions can be imaged, sometimes revealing even novel effects such as multifrequential oscillations^[^ ^179^ ^]^ or how surface structure, defects and metal/oxide interfaces steer kinetic phase transitions.^[^ ^183^, ^184^, ^185^, ^186^, ^187^, ^188^, ^189^, ^190^, ^191^, ^192^ ^]^ As a downside, PEEM is typically limited to pressures up to ≈10^−4^ mbar.

**Figure 6:**
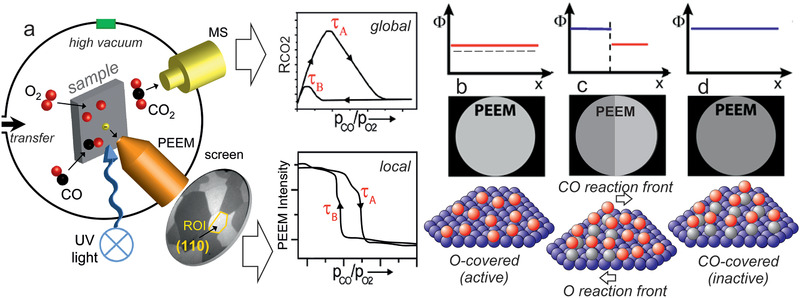
a) Illustration of operando photoemission electron microscopy (PEEM) an*d kinetics by imaging*:^[^ ^97^, ^185^, ^186^, ^187^ ^]^ a) an ongoing catalytic reaction on a structurally‐heterogeneous sample, for example, a polycrystalline Pd foil, is monitored simultaneously by PEEM and MS. Local data obtained from the intensity analysis of PEEM video‐sequences for each individual (hkl)‐domain (µm‐scale) are compared with global (averaged) MS data. The selected region of interest (ROI) may be an individual µm‐sized domain or an oxide‐supported metallic agglomerate. b–d) Schematical comparison of the PEEM intensity of a clean (dashed line), O‐covered and CO‐covered Pd surface, enabling to differentiate catalytically active from inactive regions, as well as to monitor kinetic transitions. See the text for details. a) Adapted with permission.^[^ ^189^ ^]^ Copyright 2013, Springer.

In PEEM, a magnified image of the sample surface is created by photoelectrons emitted upon UV or X‐ray illumination (lateral resolution of the used lab‐instrument: ≈1 µm; **Figure** **6**a). PEEM is a parallel imaging method, so that information on the entire field‐of‐view is simultaneously recorded, in contrast to scanning microscopies. The PEEM image intensity is related to the local work function. Variations may be due to different surface structures (e.g., (100), (110), (111) thus having different intensities), but also adsorbates/reactants change the work function (Figure 6b–d).^[^ ^185^, ^186^, ^187^, ^188^, ^189^, ^190^, ^191^, ^192^, ^193^, ^194^, ^195^, ^196^ ^]^ The local evolution of surface coverage is hence directly reflected by the local PEEM image intensity. For example, during catalytic CO oxidation on polycrystalline Pd, the catalytically active (oxygen covered; low work function; Figure 6b) surface regions appear bright on the PEEM screen and can be easily differentiated from the inactive (CO covered; high work function; Figure 6d) surface, which looks dark.^[^ ^98^ ^]^ When reaction parameters are changed (partial pressures or temperature; Figure 6a), local kinetic transitions between the active and inactive state can be observed and analyzed (Figure 6a,c). This provides a direct evaluation of local reaction kinetics under various experimental conditions just by imaging, termed *local kinetics by imaging*.^[^ ^86^, ^97^, ^185^, ^186^, ^187^ ^]^ Such spatially‐resolved sampling of reaction kinetics is more informative than usual global kinetic measurements, performed by GC or MS measurements, which give values that are averaged over the entire sample (compare MS and PEEM data in Figure 6a), smearing out the heterogeneity of the catalytic surface. Nevertheless, the averaging MS data are still valuable for comparison with other averaging measurements.

Clearly, PEEM is most powerful when applied to structurally‐heterogeneous surfaces, such as the polycrystalline Pd foil shown in Figure 6a. Indeed, the foil represents a surface structure library^[^ ^97^, ^98^, ^186^, ^187^ ^]^ as it consists of many surface domains with different crystallographic orientations and different sizes (Figure 6a), the orientation of which can be precisely determined by electron backscatter diffraction (EBSD). When combined with PEEM, the main advantage of such a heterogeneous, but well‐defined, model system is the possibility to examine, in “one video” the inherent surface properties of many different crystallographic orientations simultaneously under identical reaction conditions.^[^ ^185^, ^186^, ^187^ ^]^ For example, this allowed to determine the structure‐sensitivity of various catalytic phenomena: of catalytic ignition in CO oxidation on Pd and Pt foil,^[^ ^98^ ^]^ of the oscillation frequency^[^ ^179^ ^]^ and the H front propagation velocity in H_2_ oxidation on (oxidized) Rh foil.^[^ ^86^ ^]^ Below, we will focus on applying PEEM to mesoscopic Pd and Rh particles (1–200 µm in size) supported on planar thin oxide films, representing particle size libraries, enabling to simultaneously determine and directly compare the catalytic properties of particles of different sizes.^[^ ^183^, ^184^ ^]^

The sample for PEEM is a 10 × 10 mm^2^ polished foil of 0.2 mm thickness, which was flame annealed in air and further cleaned by repeated cycles of sputtering with Ar^+^ ions at 1 keV at room temperature and consecutive annealing to 800 °C for 30 min, until XPS indicated a clean surface. In the study below, a deuterium discharge UV lamp was utilized (≈190 nm; photon energy ≈ 6.5 eV). The local pressure of reactants is up to 10^−4^ mbar, as differential pumping and two in‐line apertures along the photoelectron trajectory reduce the pressure inside the PEEM to below 10^−7^ mbar. PEEM images are recorded by a high‐speed CCD camera and magnification is calibrated by comparison with optical or electron microscopy images of the sample. The recorded PEEM images/videos are complemented by area‐averaged MS‐data, obtained simultaneously by a quadrupole mass spectrometer placed in the vicinity of the sample (Figure 6a). For reviews, single crystal studies and a recent NAP‐PEEM variant, which enables high resolution (<100 nm) imaging around 1 mbar from 150–1200 K, refer to.^[^ ^95^, ^187^, ^193^, ^194^, ^195^, ^196^, ^197^, ^198^ ^]^

### 3.5 Density Functional Theory

The developments in operando methodology enable to relate a specific catalyst state to its performance under reactive conditions. However, in many cases the interpretation and verification of the obtained experimental spectra/images/patterns relies on theoretical support, for example, by simulating test structures of clusters and nanoparticles and their corresponding spectra, calculating adsorption and reaction energies or micro‐kinetic modelling.^[^ ^33^, ^98^, ^159^, ^199^, ^200^, ^201^, ^202^, ^203^, ^204^, ^205^, ^206^, ^207^, ^208^, ^209^, ^210^ ^]^ Based on its important role in catalysis, DFT is explicitly mentioned here, but without any technical details (which can be found in the references). Clearly, if calculations are performed for realistic gas pressures (coverage) and temperatures, they are even more relevant for operando studies.

## 4 Results and Discussion

Five representative examples of operando studies of nanocatalysts are discussed in detail in the following, covering the range from clusters via nanoparticles to meso‐scale aggregates, from a few via hundreds/thousands to a quadrillion of atoms. The first example describes the smallest entities, that is size‐controlled Au_38_ clusters supported on CeO_2_, and illustrates how their dynamic structure changes can be followed in situ by XANES/EXAFS and under reaction conditions by DRIFTS. Ligand as well as atom mobility were observed during CO oxidation. The second study deals with supported bimetallic CuNi nanoparticles, employed for methane reforming and examined by operando NAP‐XPS, ex situ HRTEM/EDX/FTIR, and DFT. Ni surface segregation, induced by CH_x_ deposits, was directly observed in the course of the reaction. Switching from area‐averaging spectroscopy to surface microscopy, the third example shows how the ongoing CO oxidation on Pd/ZrO_2_ catalysts is directly visualized by PEEM, and how the local kinetics can be deduced by imaging kinetic phase transitions. PEEM of H_2_ oxidation on Rh/ZrO_2_ reveals the “inverse role” of oxygen in this reaction. Operando PEEM directly proves that the nano‐scale metal/oxide interface steers the behavior of an entire meso‐scale particle. Finally, further developments and time‐resolved spectroscopy are illustrated by CO oxidation on Co_3_O_4_ and MSR on Pd_2_Ga/Ga_2_O_3_. All studies are paralleled by simultaneous product analysis, which is key to the operando approach. The relevant adsorbed species can clearly be not observed ex situ, but also post‐reaction analysis of structures, surface composition (alloys, intermetallics), and oxidation state would deliver rather indirect and sometimes ambiguous results.

### 4.1 Atomically‐Precise Au_38_ Clusters Supported by CeO_2_: Intracluster Restructuring and Atom Mobility During CO Oxidation

As mentioned, area‐averaging operando spectroscopy (e.g., XAS, NAP‐XPS, IR) is very powerful, but unable to account for catalyst heterogeneity (dispersion in particle size, shape, surface structure, composition, distribution on the support, etc.). One strategy to circumvent this limitation is to prepare structurally‐homogeneous monodisperse supported clusters/nanoparticles of a specific size. Previous work along these lines mostly focused on metal clusters with up to about 100 atoms, representing the so‐called non‐scalable regime, in which nearly every atom counts. Three major synthesis routes were followed: i) clusters prepared and mass‐selected in the gas phase, prior to “soft‐landing” on suitable supports,^[^ ^29^, ^30^, ^31^, ^211^, ^212^, ^213^, ^214^ ^]^ ii) colloidal size‐ and shape‐controlled nanoparticles impregnated on various supports,^[^ ^7^, ^24^, ^215^, ^216^, ^217^ ^]^ and iii) wet‐chemically prepared ligand‐protected clusters in solution,^[^ ^218^, ^219^, ^220^, ^221^, ^222^, ^223^, ^224^ ^]^ “size‐purified” by size‐exclusion‐chromatography (SEC) or gel‐electrophoresis prior to deposition on a support. Although the initial state of such monodisperse nanoparticles is well‐defined, they typically undergo structural rearrangements and/or atom mobility during catalysis, which is why operando characterization is once more inevitable to understand their catalytic performance. Herein, the focus is on (iii), that is, thiolate (SC_2_H_4_Ph) protected Au clusters, and monitoring their structural rearrangements. Whereas catalysis on Au was a niche in the 1970s,^[^ ^225^ ^]^ since the 1990ies there is a second “gold rush”,^[^ ^226^, ^227^, ^228^, ^229^ ^]^ realizing the opportunities and potential benefits of catalysis by gold.^[^ ^8^, ^230^, ^231^, ^232^, ^233^ ^]^

**Figure 7:**
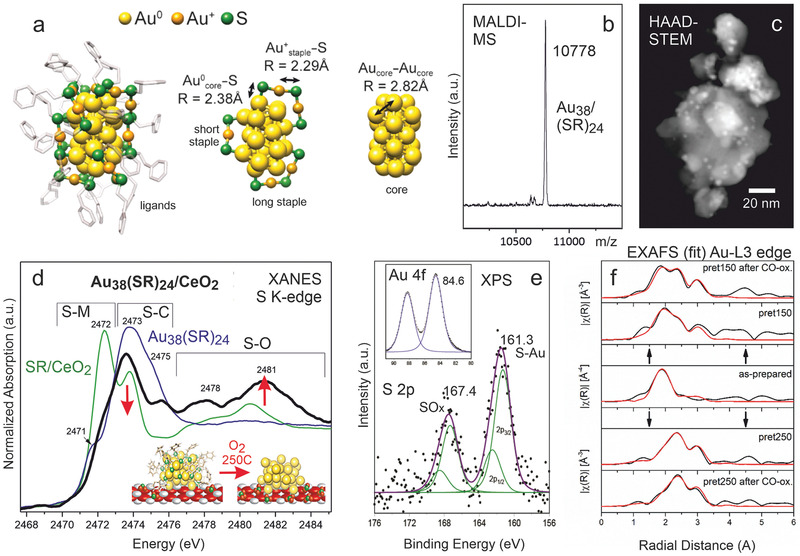
Activation of Au_38_(SR)_24_ nanoclusters supported on CeO_2_ (nominal Au loading 1.4 wt%): a) Initial structure with characteristic distances and b) corresponding MALDI‐MS spectrum. c) HAADF‐STEM images after activation in O_2_ at 250 °C and CO oxidation. d) XANES S K‐edge spectra of the unsupported clusters, the thiol ligand supported on CeO_2_ and the Au_38_/CeO_2_ catalysts (after deposition). The red arrows illustrate the changes upon oxidation at 150 °C and 250 °C. e) S 2p and Au 4f (inset) core level XPS spectra after activation at 150 °C. f) analysis and fit (red) of in situ EXAFS spectra of the as‐prepared catalyst, after activation (at 150 or 250 °C) and post‐reaction. a,b,c,f) Adapted with permission.^[^ ^27^ ^]^ Copyright 2020, American Chemical Society. d,e) Adapted with permission.^[^ ^99^ ^]^ Copyright 2018, Wiley VCH.

The high affinity between Au and S, well‐know from self‐assembled monolayers, enables that ligand‐protected Au clusters can be prepared with atomic precision, meaning that the number of Au atoms in a cluster is exactly controlled.^[^ ^219^, ^220^, ^234^ ^]^ As an example, **Figure** **7**a shows the Au_38_(SC_2_H_4_Ph)_24_ structure, as derived from X‐ray crystallography^[^ ^220^ ^]^ and EXAFS,^[^ ^234^ ^]^ consisting of a symmetric biicosahedral Au_23_ core, which is protected by three monomeric “short” staples (SR‐Au‐SR) and six dimeric “long” staples (SR‐Au‐SR‐Au‐SR), with three characteristic distances indicated: Au‐Au between neighboring Au (core) atoms and two different Au‐S bonds (Au°_core_‐S and Au^+^_staple_‐S). The exact Au_38_(SC_2_H_4_Ph)_24_ cluster composition and size of ≈2.5nm were confirmed by matrix‐assisted laser desorption/ionization (MALDI)‐MS and high‐angle annular dark‐field (HAADF)‐STEM, respectively (Figure 7b,c).

Similarly, clusters with 10, 11, 15, 18, 20, 22, 24, 25, 28, 29, 33, 36, 38, 39, 40, …, 102, 107, 130, 144, 187, etc. Au atoms can be prepared and Au_25_(SC_2_H_4_Ph)_18_, Au_38_(SC_2_H_4_Ph)_24_ and Au_144_(SC_2_H_4_Ph)_60_ are exemplarily mentioned here.^[^ ^27^, ^99^, ^175^, ^235^, ^236^, ^237^, ^238^ ^]^ The number and configuration/type of ligands/staples typically varies, enabling to tune the physical properties and catalytic performance of the clusters. Once a pure solution of monodisperse Au*_x_* (SR)*_y_* clusters has been obtained, it can be used for standard impregnation of supporting oxides (e.g., CeO_2_, TiO_2_, Al_2_O_3,_ SiO_2_).

The cluster size and structure are typically maintained upon deposition on a support. However, except for some specific applications (HDS, Fischer–Tropsch, selective hydrogenation), the sulphur in the ligands is typically a catalyst poison^[^ ^239^ ^]^ and must be removed before catalytic reactions can take place.^[^ ^99^, ^236^, ^237^ ^]^ In situ XANES and EXAFS at the Au edge, detecting diminishing Au‐S bonds upon after oxidative (5% O_2_/He) pretreatment,^[^ ^240^ ^]^ suggested that the thiolate ligands are mostly converted to (gaseous) SO_2_, CO_2_, and H_2_O.

However, follow‐up ex situ S K‐edge XANES studies of Au_38_(SC_2_H_4_Ph)_24_/CeO_2_ (Figure 7d) revealed that already upon deposition the sulfur ligands partly migrate from the Au clusters to the (ceria or alumina) support (to a lesser extent; reduced S–C peak at 2473 eV and weak S–Ce shoulder at 2472 eV), but specifically upon oxidative pre‐treatment at 150 and 250 °C (strongly reduced S–C peak at 2473 eV),^[^ ^99^ ^]^ finally forming mostly sulfate on the support (strongly increased SO_4_ peak at 2481 eV). STEM‐HAADF with EDX analysis revealed that S‐species are still located near the Au clusters (with no S detected in between them). XPS spectra (Figure 7e) of Au 4f and S 2p regions acquired after 150°C pretreatment (S 2p quite noisy due to the low cluster loading) confirmed the onset of partial ligand removal (weak S‐Au bonds at 161.3 eV and SO*_x_* at 167.4 eV).

Accordingly, oxidative cluster activation leads to clean(er) Au cluster surfaces, but also modifies the surrounding oxide support by sulphur‐containing species. For Au*_x_* (SC_2_H_4_Ph)*_y_*/CeO_2_^[^ ^238^, ^241^ ^]^ this leads to catalysts active in CO oxidation, but the activation process is even more complex, as discussed below. In contrast, TiO_2_ supported Au clusters remain inactive even after extended activation.^[^ ^238^, ^242^ ^]^ It seems that the SO*_x_* groups poison the interfacial Au/TiO_2_ sites, which are responsible for oxygen activation. The interface/lattice oxygen is required to react with CO adsorbed on Au,^[^ ^241^ ^]^ according to a Mars van Krevelen (MvK) mechanisms. Further studies of TiO_2_ supported Au clusters are need to clarify this issue.

Returning to Au_38_(SC_2_H_4_Ph)_24_/CeO_2_, the picture of Au cluster activation by oxidative ligand removal was further refined by in situ Au L_3_‐edge EXAFS (Figure 7f), again complemented by ex situ STEM‐HAADF and XPS.^[^ ^27^ ^]^ The changes in cluster structure upon pretreatment are reflected by two EXAFS parameters, *R* (distance) and *N* (coordination number of neighboring equivalent atoms), but only the latter is discussed here. Upon oxidative pre‐treatment at 150 or 250 °C, which increasingly “remove” thiol ligands, the number of nearest equivalent Au neighbors (*N*_Au–Au_) increased (from initially 2.59 to 5.08 and 8.09, respectively). However, HRTEM did not show any cluster agglomeration and the coordination numbers of Au°_core_‐S and Au^+^_staple_‐S also did not simply continuously decrease, altogether indicating a more complex behavior.^[^ ^27^ ^]^

**Figure 8:**
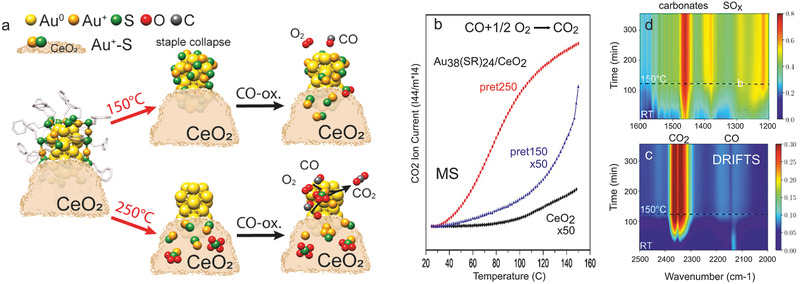
Dynamic structure changes of Au_38_(SR)_24_ clusters on CeO_2_ upon activation and CO oxidation reaction (derived from in situ EXAFS, ex situ HAAD‐STEM, XPS) and operando DRIFTS: a) schematics. b) MS analysis of CO oxidation on Au_38_(SR)_24_/CeO_2_ and (pure) CeO_2_ (3.3% CO, 7% O_2,_ 89.7% He, total flow: 60 mL min^−1^, ramp: 5 °C min^−1^; CO_2_ traces were normalized to the catalyst mass and the He signal). Operando DRIFTS of Au_38_/CeO_2_ activated at 250 °C, from room temperature to 150 °C: c) 2500–2000 cm^−1^ and d) 1600–1200 cm^−1^. Adapted with permission.^[^ ^27^ ^]^ Copyright 2020, American Chemical Society.

When Au_38_(SR)_24_/CeO_2_ was pre‐treated at 150 °C, N Au^+^_staple_‐S strongly decreased (from 0.91 to 0.12), but N Au°_core_‐S unexpectedly increased (from 0.66 to 0.82), suggesting a collapse of the remaining staples, creating new Au°_core_‐S bonds, in addition to Au–Au. Thus, the hydrocarbon backbone was oxidized, but the S remaining on Au then acts as catalyst poison. Upon oxygen pre‐treatment at 250 °C, N Au°_core_‐S became almost zero, so that S is almost completely removed from the cluster core, producing bare Au surfaces. Note that Au^+^‐S configurations were still present (N Au^+^_staple_‐S of 0.45), which must be located on the support, as one can exclude that staples that collapse at 150 °C are reestablished at 250 °C. The deduced evolution of the Au_38_(SR)_24_/CeO_2_ cluster structure upon treatment at 150 or 250 °C is depicted in **Figure** **8**a. Turning to catalytic CO oxidation monitored by MS, Figure 8b compares differently pretreated Au_38_(SR)_24_/CeO_2_ with the pure CeO_2_ support. The as‐prepared Au_38_(SR)_24_/CeO_2_ catalyst holding intact ligands was nearly inactive. Au_38_(SR)_24_/CeO_2_ pretreated at 150 °C showed only minute activity even at the maximum reaction temperature of 150 °C (note the ×50 scale). This confirms the presence of collapsed staples derived from EXAFS, explaining why the S‐poisoned Au atoms were inaccessible for the reactants CO and O_2_. However, after activation at 250 °C, Au_38_(SR)_24_/CeO_2_ became 50× more active, with activity setting in below 40 °C (which points to clean Au surfaces, as Au^+^‐S species are inactive).

In situ EXAFS (Figure 7f) and operando DRIFTS of Au_38_/CeO_2_ acquired during CO oxidation (Figure 8c,d) revealed further reaction‐induced continuous structural changes via CO‐induced Au and O‐induced S migration to the support. For the active catalyst pretreated at 250 °C (pret250), the Au core structure was preserved upon reaction, evident from only slightly reduced Au‐Au coordination numbers in EXAFS fitting. Interestingly, N Au^+^_staple_‐S decreased (from 0.45 to 0.31), while N Au°_core_‐S increased (from 0.04 to 0.15) during the reaction. As S migration back to the cluster is unlikely, this suggests that (isolated) Au^+^_staple_‐S dissociated and/or agglomerated to Au°_core_‐S on the support. Alternatively, more Au atoms may diffuse to the support and merge with S. However, the two Au‐S species will not contribute to catalytic activity, as S is a poison. A decrease of N Au–Au (from 8.09 to 7.04) additionally points to Au atom migration (redispersion) under reaction conditions, which may result from CO‐induced Au atom mobility, as also observed for Pt, Pd, or Cu.^[^ ^24^, ^25^, ^26^, ^27^, ^28^ ^]^ For the less active catalyst pretreated at 150 °C (pret150) with S‐poisoned Au surfaces, activity did not increase significantly, but some S moved to the support during the reaction (decreasing N Au°_core_‐S (from 0.82 to 0.17) and increasing N Au–Au (from 5.08 to 6.98)), merging with mobilized Au atoms (increasing N Au^+^_staple_‐S (from 0.12 to 0.44)). The reaction‐induced evolution of the Au_38_(SR)_24_/CeO_2_ catalyst is also depicted in Figure 8a.

After characterizing catalyst structure and composition, the adsorbed species were monitored by operando DRIFTS during CO oxidation from room temperature to 150 °C (Figure 8c,d). For the highly active Au_38_(SR)_24_/CeO_2_ (pret 250 °C), the gas‐phase bands at 2300–2400 cm^−1^ (Figure 8c) indicated CO_2_ formation already at room temperature, in line with MS (Figure 8b). Adsorbed CO on Au^0^ (2130 cm^−1^) was observed up to 120 °C (note that any Au surface atom on a cluster is low‐coordinated).^[^ ^231^ ^]^ Monodentate carbonates (1468 cm^−1^)^[^ ^71^, ^162^, ^243^ ^]^ developed in the lower wavenumber region (Figure 8d), likely located on ceria or close to the metal/oxide interface. Increasing IR bands characterizing surface SO_3_ (or S_2_O_7_^−2^) and SO_4_ (S=O, S—O—Ce) were also detected,^[^ ^244^ ^]^ whereas bulk SO_4_ was absent (missing bands at 1196, 1128 cm^−1^). Accordingly, operando DRIFTS directly monitored the further ligand/sulfur migration to the support during reaction. These changes were additionally confirmed by ex situ STEM‐HAADF, imaging Au nanoclusters of ≈2.5nm size in all states, and by XPS (Au4f binding energy shifts were in line with the various states of ligand removal).

Apparently, even for the “seemingly simple” Au_38_/CeO_2_ catalyst the involved cluster chemistry is quite complex, which may explain diverging reports on the effect of pretreatment on catalytic activity, as the extent of ligand collapse and migration depends on the applied conditions. Only operando spectroscopy allows straightforward conclusions. This not just holds true for the discussed thiol ligands, as similar complex relationships between synthesis/activation/reaction are typically observed also for S‐free systems, for example, for Pt/CeO_2_ (effects of precursor, synthesis route and activation) or Pd/MgO (size effects).^[^ ^69^, ^243^, ^245^, ^246^ ^]^ The monodispersity of the Au_38_ clusters is a big advantage for spectra interpretation, as size‐dispersion can be largely ruled out.

Combining operando methods with atomically‐precise clusters, further improvements of nanocatalyst functionality seem possible. For example, Au clusters can be doped with single atoms of Pt, Pd, Cu, or Ag with atomic precision, because the exact adatom position within the Au cluster (in the core center, on the core surface, in the staples) can be controlled by the dopant type, number, pretreatment and reaction conditions.^[^ ^224^, ^242^, ^247^, ^248^, ^249^, ^250^ ^]^ This creates a particularly promising strategy towards bimetallic catalysis and single site catalysis (site isolation).^[^ ^10^, ^11^, ^16^, ^20^, ^242^, ^251^ ^]^ For example, operando DRIFTS has been applied to monitor the reaction‐induced mobility of Pd dopant atoms within PdAu_24_ clusters, moving from the cluster core to the cluster surface and yielding a Pd single‐site catalyst (Pd‐Pd bonds were also absent in EXAFS).^[^ ^242a^ ^]^ In supported Ag*_x_* Au_25‐_ *_x_* (*x* = 6‐8), single Ag atoms were initially dispersed over the nanocluster surface, still holding thiol ligands and surrounded by Au‐containing staples.^[^ ^242b^ ^]^ Upon CO oxidation, the ligands were removed, creating a better defined AgAu alloy and small Ag‐Ag patches, as corroborated by EXAFS and DRIFTS. Further routes towards nanocluster engineering include other metal clusters (e.g., Co,^[^ ^223^ ^]^ Ni,^[^ ^255^ ^]^ Ag^[^ ^256^ ^]^), metal‐metal,^[^ ^257^ ^]^ and ligand‐ligand^[^ ^222^, ^258^ ^]^ exchange reactions.

### 4.2 Bimetallic CuNi Nanoparticles Supported by ZrO_2_: CH_x_‐Induced Ni Surface Segregation during Methane Decomposition

The monodisperse cluster catalysts discussed in the previous section represent well‐suited systems for systematic studies of size effects. However, at the cost of quite complex synthesis/purification involving thiols, which may limit straightforward technological applications. For technological catalysts, there are well‐established simpler routes such as incipient wetness impregnation, but their downside is that a range of different particle sizes is present. Nevertheless, well‐controlled synthesis is able to produce narrow size/shape distributions.^[^ ^7^, ^17^, ^215^, ^216^, ^217^ ^]^ In the following, the application of Ni and CuNi nanoparticles supported on zirconia (ZrO_2_) for methane reforming is discussed,^[^ ^259^, ^260^, ^261^, ^262^ ^]^ with operando NAP‐XPS studies revealing reaction‐induced surface composition changes.

**Figure 9:**
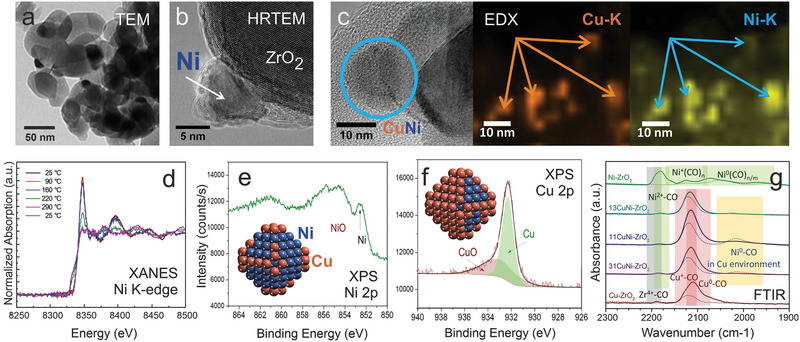
a) TEM and b) HRTEM images of Ni/ZrO_2_. c) HRTEM and elemental EDX mapping (K edge) of 1:1 CuNi/ZrO_2_. The encircled nanoparticle contained both Cu and Ni. d) In situ XANES K‐edge spectra during reduction in H_2_. e) Ni 2p_3/2_ and f) Cu 2p_3/2_ in situ XPS of 1:3 CuNi/ZrO_2_ in 0.25 mbar H_2_ at 400°C (*E*_kin_ of 150 eV). g) infrared spectra of reduced catalysts in 5 mbar CO pressure (solid lines) and after evacuation (dashed lines) at room temperature. a,b) Adapted with permission.^[^ ^263^ ^]^ Copyright 2016, Elsevier. c,e,f) Adapted with permission.^[^ ^264^ ^]^ Copyright 2015, The Royal Society of Chemistry. d,g) Adapted with permission.^[^ ^265^ ^]^ Copyright 2013, Springer.

For Ni/ZrO_2_, (HR)TEM and XRD analysis indicated monoclinic ZrO_2_ grains (50–100 nm in size) and face centered cubic (*fcc*) Ni nanoparticles of around 10 nm size (with a disordered NiO shell). **Figure** **9**a,b show TEM overview and lattice resolution images, respectively.

In a simplified picture, Methane Steam Reforming (MSR) can be envisioned by two basic processes: i) CH_4_ decomposition on Ni, leading to surface carbon and hydrogen (CH_4_ ⇄ C + 2 H_2;_ 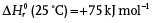) and ii) carbon gasification by water, with the latter activated at the metal/oxide interface (C + H_2_O → CO + H_2;_ 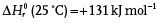).

Operando FTIR (CH_4_:H_2_O = 1:3 up to 400 °C at 1.2 bar with simultaneous MS analysis) proved CH_4_ and H_2_O activation via the presence of adsorbed CH_3_/CH_2_ and OH species, respectively.^[^ ^266^ ^]^ Clearly, both processes (i) and (ii) must be balanced so that carbon accumulation on Ni is avoided. Indeed, this turns out to be the major challenge of the process, as carbon filaments (whiskers) grown on the Ni nanoparticles lead to catalyst deactivation and reactor clogging by coking.^[^ ^267^ ^]^ Two strategies may therefore be employed to suppress coking: i) reducing the (local) rate of methane decomposition by decorating low‐coordinated sites on the Ni nanoparticle by less‐active metals (e.g., Au, Sn, Cu), leading to bimetallic nanoparticles,^[^ ^264^, ^268^ ^]^ or ii) increasing the rate of carbon removal by faster water activation at the metal/oxide interface (or of CO_2_ for methane dry reforming (MDR)), for example, by using more active mixed‐oxide supports.^[^ ^263^ ^]^ In the following, both strategies are illustrated and their success finally compared.

Bimetallic CuNi nanoparticles on ZrO_2_ were prepared by standard impregnation.^[^ ^264^, ^268^ ^]^ For an octahedral Ni nanoparticle of about 6 nm size (3235 atoms; 1020 surface atoms), the decoration of edges and corners with Cu would require a Cu:Ni ratio of ≈1:3 (cf. the schematic illustration in Figure 9e). However, simple “target compositions” typically do not work in synthesis (for the reasons described below), which is why three different nominal Cu:Ni compositions (1:3, 1:1, 3:1 in wt%.) were prepared on ZrO_2_ (all with 5 wt% loading; for XPS also samples with 50 wt% were used to reduce charging). Note that the ratios refer to wt.%, but as the atomic masses of Cu (63.5) and Ni (58.7) are similar, they approximate atomic ratios.

**Figure 10:**
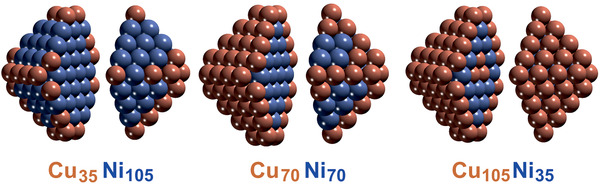
DFT‐derived surface and interior composition of bimetallic nanoparticles: Cu_35_Ni_105_ (1:3)_,_ Cu_70_Ni_70_ (1:1) and Cu_105_Ni_35_ (3:1), with optimized chemical ordering. Color code: Cu—redwood, Ni—blue. Adapted with permission Copyright 2017, Elsevier.

After catalyst synthesis and in situ oxidation/reduction the CuNi nanoparticles were characterized by HRTEM/EDX, XRD, XANES, NAP‐XPS, and FTIR (using CO as probe molecule).^[^ ^264^, ^265^, ^268^ ^]^ EDX mapping (Figure 9c) of CuNi 1:1 proved that Cu and Ni were present at the same location, indicating bimetallic particles. In situ XANES (Ni K edge) during H_2_ reduction showed that in the bimetallic samples Ni was reduced at 220 °C, 160 °C lower than Ni/ZrO_2_, once more pointing to bimetallics.^[^ ^265^ ^]^ However, the location of Cu and Ni within a nanoparticle is unknown, but this can be answered by surface‐sensitive methods. First, in situ NAP‐XPS in hydrogen indicated Ni/NiO and stronger Cu signals, suggesting a Cu‐rich surface (Figure 9e,f). Second, FTIR of the adsorption of the probe molecule CO was used to characterize the surface. Figure 9g compares CO spectra of Ni/ZrO_2_, Cu/ZrO_2_ and of the three bimetallic catalyst, proving that after synthesis all bimetallic particles had a Cu‐rich surface (cf. the model in Figure 9f), even when the overall nanoparticle composition was rich in Ni. The Cu‐enrichment was rationalized by DFT (Figure 10),^[^ ^268^, ^269^ ^]^ which revealed that in all studied compositions Cu segregation to the surface is energetically favorable, with Cu preferentially occupying corner and edge sites. Only when the number of Cu atoms is too small to occupy all surface sites, Ni is present on the particle terraces. In **Figure** **10**, this is illustrated for nanoparticles with 140 atoms (100 surface atoms) for Cu:Ni compositions of 1:3, 1:1, and 3:1 (for larger particles see ref. ^[^ ^268^ ^]^).

**Figure 11:**
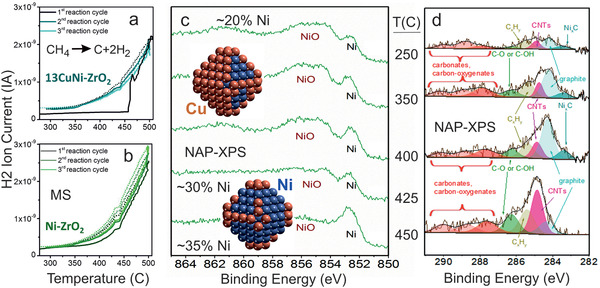
Hydrogen evolution and surface composition changes during methane decomposition. MS traces on a) 1:3 CuNi/ZrO_2_ and b) Ni/ZrO_2_. Catalysts were reduced at 400 °C, followed by 3 heating (solid lines) / cooling (dashed lines) cycles (5 °C min^−1^) between 300 and 500 °C in CH_4_/Ar. c) Ni 2p_3/2_ and d) C1s XPS of 1:3 CuNi/ZrO_2_ in 0.25 mbar CH_4_ upon heating from 250 to 450 °C (*E*_kin_ of 150 eV; incident photon energies were 1100, 1010, and 425 eV for Cu, Ni, and C, respectively). The models of Cu_70_Ni_70_ in (c) illustrate the Ni segregation to the surface, induced by CH*_x_* groups detected in d). Adapted with permission.^[^ ^264^ ^]^ Copyright 2015, The Royal Society of Chemistry.

In the next step, supported 1:3 bimetallic CuNi nanoparticles were used for catalytic temperature‐dependent CH_4_ decomposition (H_2_ evolution; cycling up to 500 °C), with synchrotron‐based NAP‐XPS employed for operando characterization.^[^ ^264^ ^]^ In the first reaction cycle (**Figure** **11**a), the catalyst was inactive at low temperature, but became active above 425 °C, and maintained its activity upon cool‐down and repeated cycles. As evident from operando NAP‐XPS (Figure 11c), the reactivity onset of CuNi/ZrO_2_ can be explained by Ni segregation to the nanoparticle surface. This agrees with the observation that Ni/ZrO_2_ was already active during the first heat‐up (Figure 11b). At first, one may suspect that the segregation was CO‐induced, as the CO—Ni bond is stronger than the CO—Cu bond (desorption temperatures of 150 and −130 °C in ultrahigh vacuum, respectively). However, NAP‐XPS also detected the simultaneous temperature‐dependent formation of various carbonaceous species at the particle surface (Figure 11d). DFT calculations indicated that indeed the adsorption of CH_x_ groups (*x* = 0–3) provided the driving force for the segregation of Ni atoms to the surface, with CH_3_ yielding the lowest and C the highest stabilization.^[^ ^268^ ^]^

**Figure 12:**
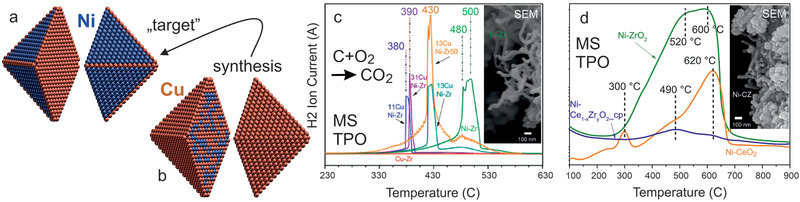
Model of the “ideal” CuNi nanoparticle (2435 Cu atoms and 840 Ni atoms, 3275 atoms in total). a) target structure under reaction conditions and b) as‐synthesized. Temperature programmed oxidation (CO_2_ (m/z 44) trace upon heating in 20 vol% O_2_/Ar; 5 K min^−1^) after methane decomposition on (c) CuNi/ZrO_2_ and Ni/ZrO_2_ catalysts, and (d) after reforming on Ni/ZrO_2_, Ni/CeO_2_ and Ni/Ce_1‐_ *_x_* Zr*_x_* O_2_ (24 h). The insets in (c) and (d) are post‐reaction SEM images displaying different amounts of carbon filaments. c) Adapted with permission.^[^ ^264^ ^]^ Copyright 2015, The Royal Society of Chemistry. d) Adapted with permission.^[^ ^263^ ^]^ Copyright 2016, Elsevier.

Combined operando spectroscopic and theoretical studies thus indicate that the CuNi/ZrO_2_ catalyst adopts a Ni‐rich surface under reaction conditions, independent of the initial surface composition after synthesis and pretreatment. Ex situ post‐reaction studies would have detected more Ni and carbon on the surface, but a correlation between them and their relation to catalytic activity would only be speculative. Based on these findings one needs to revise the synthetic strategy towards an “ideal” CuNi alloy particle with improved coke resistance. Independent of the overall initial nominal composition, a Cu‐rich surface is obtained after synthesis and activation (oxidation/reduction), due to the lower surface energy of Cu especially at edges and corners (Figures 9 and 10). However, as Ni segregates to the surface during reaction (Figure 11), when the amount of Ni in the nanoparticle is sufficient to create a (nearly) complete Ni surface, the coking resistance is lost (due to the absence of surface Cu). Consequently, an “ideal” nanoparticle should have edges and corners from Cu, and terraces of Ni (**Figure** **12**a). Furthermore, the nanoparticle interior must be Cu, so that Ni surface segregation is limited. Thus, one should synthesize a Ni‐modified Cu particle (Figure 12b) rather than a Cu‐modified Ni particle! Accordingly, a ≈6 nm large octahedral CuNi nanoparticle must have 2435 Cu atoms and 840 Ni atoms, so overall 3275 atoms. In this case, the segregated Ni atoms can just cover the nanoparticle terraces, whereas the Cu atoms are located at the 180 edge/corner and 2255 interior sites (Figure 12a).

According to DFT, in the global minimum structure (0 K) all Ni atoms would indeed be located in the nanoparticle interior. For surface segregation of 840 Ni atoms an energy of 166 eV is required.^[^ ^268^ ^]^ This seems very large, but the energy is delivered by the adsorbed CH*_x_* groups that were detected by NAP‐XPS. Even 332 weakly adsorbed CH_3_ groups at 0.4 ML terrace coverage provide sufficient energy for Ni segregation and for more strongly adsorbed CH_2_, CH, or carbon, significantly lower coverage is necessary.

The “ideal” Cu_2435_Ni_840_ particle has a 3:1 Cu:Ni composition, which was included in the original synthesis, and in fact it showed reduced coking. As evident from temperature‐programmed oxidation (Figure 12c), the amount of coke was not only about half, but it was reoxidized at ≈100 °C lower temperature. Consequently, the coke formed on the bimetallics was less‐stable than that formed on Ni/ZrO_2_. In turn, the improved coking resistance of 3:1 Cu:Ni goes along with a five‐fold reduction in H_2_ yield (resp. rate per gram of catalyst), whereas the rate per gram of Ni remained nearly the same.^[^ ^264^ ^]^ Thus, when the most active edge/corner/step sites of Ni are decorated/replaced by Cu, coking is significantly reduced, but unfortunately this also reduces the overall reforming activity.

Slowing down CH_4_ decomposition seems not the best strategy, which is why the acceleration of water activation (for MSR or of CO_2_ for MDR) at the metal/oxide interface may be more successful in obtaining the desired balance of carbon‐formation and carbon‐removal. Mixed ceria‐zirconia, prepared by co‐precipitation and leading to higher surface area than that of pure ZrO_2_ or CeO_2_ (91, 37, and 56 m^2^ g^−1^, respectively), was thus employed as more active support for Ni nanoparticles.^[^ ^263^ ^]^ The support was a mixture of a cubic ceria‐rich mixed oxide (Ce:Zr = 9:1) and tetragonal zirconia‐rich mixed oxide (Ce:Zr = 1:9). The activity of the Ni/Ce_1‐_ *_x_* Zr*_x_* O_2_ catalyst in MDR was initially even 50% higher and after 24 h similar to that of Ni/ZrO_2_, but the formation of graphitic (≈520 °C) and filamentous carbon (≈600 °C) was reduced about 100× (Figure 12d). Consequently, using a ceria‐zirconia support effectively counteracts deactivation and reactor tube blocking. Note that Ni/CeO_2_ was about half as active as Ni/ZrO_2_ and accordingly produced less coke (which is why coke production must be normalized by activity). An improved catalytic performance of mixed oxides has also been shown for CeO_2_/Co_3_O_4_ in (preferential) CO oxidation, probably resulting from the Ce‐O–Co interfacial sites.^[^ ^131^, ^270^ ^]^

The operando surface spectroscopy methods discussed so far (XAS, NAP‐XPS, IR) are quite powerful, but area‐averaging over the entire sample, so that local effects could not be differentiated. In the following section, operando surface microscopy will be employed, that allows to obtain spatially‐resolved information on the catalyst surface morphology, but especially on the reacting species.

### 4.3 Mesoscopic Pd and Rh Particles Supported by ZrO_2_: Imaging Kinetic Transitions and Long‐Range Interface Effects on Individual Particles during CO and H_2_ Oxidation

To visualize (truly “see”) ongoing catalytic reactions has been a dream of catalyst researchers, as this would answer long‐standing questions: at which sites of the catalyst does a reaction start and how does it proceed? How do reaction conditions affect the coverage with reactants and how do they interact? Which processes lead to deactivation and how can they be avoided? Significant efforts have thus been devoted to study the catalytic properties of catalyst particles by various microscopic or locally‐resolved spectroscopic/diffractive methods, for example, by electron microscopy,^[^ ^50^, ^91^, ^271^, ^272^ ^]^ nuclear magnetic resonance,^[^ ^273^ ^]^ (nano‐)infrared,^[^ ^274^ ^]^ X‐ray microscopy^[^ ^85c^ ^]^ and tomography,^[^ ^51^, ^275^, ^276^ ^]^ fluorescence,^[^ ^277^ ^]^ and plasmonic nanospectroscopy.^[^ ^278^ ^]^ Recently, photoemission electron microscopy (PEEM) directly revealed a long‐ranging communication effect of the metal/oxide interface with the internal surface sites of metal particles, as discussed below for two oxidation reactions.^[^ ^183^, ^184^ ^]^

The oxidation of CO on (supported) noble metals is a benchmark reaction, both for model and applied catalysis.^[^ ^159^, ^186^, ^279^, ^280^, ^281^, ^282^ ^]^ In addition, CO adsorption is frequently used to characterize the accessible surface sites.^[^ ^36^, ^73^, ^159^, ^162^, ^282^, ^283^ ^]^ It is well‐accepted that particle size and shape and the metal/support interface govern the catalytic performance in many cases. Sites at metal/oxide interface (colored light blue in Figure 1c), termed perimeter (adlineation) sites or (triple) phase boundary sites, are often even considered to be most active.^[^ ^39^, ^40^, ^41^, ^42^, ^43^, ^104^, ^284^, ^285^, ^286^, ^287^, ^288^, ^289^, ^290^, ^291^, ^292^ ^]^ Nevertheless, metal‐support interactions are believed to be localized, that is, to only affect reactivity of sites that are <1 nm away from the interface.

For CO oxidation, enhanced reaction rates at the interface were attributed to the reaction between CO adsorbed on the metal and oxygen weakly bound to the oxide, reported, for example, for Au/TiO_2_ or Au/CeO_2_ catalysts.^[^ ^285^, ^288^ ^]^ Low‐coordinated sites of FeO_x_ islands on Pt(111)^[^ ^286^ ^]^ may also provide active O species. However, particularly reactive sites may also be present just on the metal side of the interface, that is, on the nanoparticles, as highlighted below. Clearly, studies performed by area‐averaging methods are incapable to resolve the local interface properties of a catalyst, so that this can only be addressed indirectly (e.g., by changing the support oxide, varying the particle size and thus the relative contribution of the perimeter).

**Figure 13:**
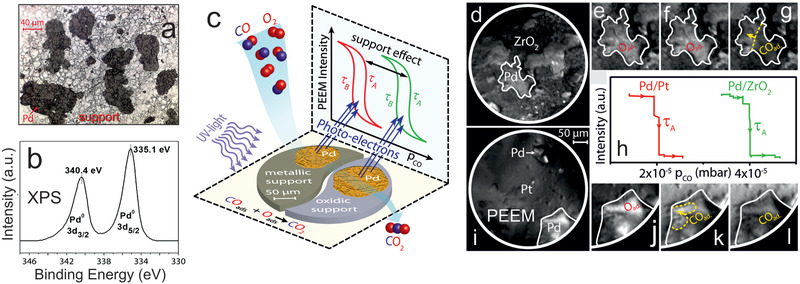
Operando surface microscopy of CO oxidation on oxide‐supported µm‐sized Pd aggregates (“Pd black”): a) optical micrograph and b) Pd 3d XPS spectrum of the clean metallic Pd particles. Comparison of the kinetic behavior of individual ZrO_2_‐supported and “quasi‐unsupported” Pd particles: c) schematic illustration of the concept (PEEM intensity versus p_CO_) and d–l) PEEM imaging of CO poisoning. Detailed conditions of PEEM images: d) field of view with several supported Pd agglomerates. e,f) Pd agglomerate marked in (d) in the active steady state (oxygen covered) upon increasing the CO pressure to 4 × 10^−5^ mbar at constant *T* = 200 °C and *p_O2_* = 1.3 × 10^−5^ mbar. g) isothermal kinetic transition (propagation of the CO front) to the inactive steady state at *p_CO_* = 4×10^−5^ mbar. h) Local PEEM intensity of the oxide supported Pd agglomerate marked in d) (green curve) and of the Pt‐supported Pd particle marked in (i) (red curve). (i) Pd agglomerates of similar size as in (d), but supported by Pt. j) Pd agglomerate marked in (i) being in the active state at the same *T* and *p*_O2_ as in (e–g) and at *p*_CO_ < 2×10^−5^ mbar. k) kinetic transition to the inactive state (CO front propagates) at *p_CO_* = 2×10^−5^ mbar. l) Pd agglomerate in the inactive state (CO covered) at *p*_CO_ > 2×10^−5^ mbar. Note that under these conditions, the oxide supported Pd agglomerate f) still remains active (oxygen covered). Adapted with permission.^[^ ^183^ ^]^ Copyright 2018, Springer Nature.

To directly examine the local catalytic activity of Pd/ZrO_2_ (spatially‐resolved) surface microscopy (PEEM) was thus used. Mesoscopic Pd powder particles (“Pd black” containing 10–200 µm sized particles) were deposited (pressed) on thin oxide films of ZrO_2_ or Al_2_O_3_ (≈1.5–2 nm thick; **Figure** **13**a).^[^ ^183^ ^]^ Non‐reducible metal oxides ZrO_2_ and Al_2_O_3_ were chosen as supports, instead of reducible metal oxides, to rule out O supply by the support (MvK). Cleaning was performed by repeated oxidation/reduction cycles, until the sample was XPS‐clean (Figure 13b). As PEEM can simultaneously image several particles of different size and shape within the field of view, this sample represents a particle size library, enabling to directly discern size effects.^[^ ^186^ ^]^

Before discussing the PEEM experiments, it is advisable to recall the Langmuir‐Hinshelwood mechanism of CO oxidation. Based on the well‐known schemes of co‐adsorbed CO and O on the catalyst surface, one is tempted to assume that in the active state there is a mixed layer of CO and O. However, even for this simple reaction it is not as simple as that, because the CO_2_ formation rate is determined by the competitive adsorption of CO and oxygen on the palladium surface. At low CO:O_2_ ratio (low *p_CO_*) the surface is covered by atomic oxygen (Figure 6b), which easily reacts with adsorbed CO; O does not hinder CO adsorption and hence the catalyst is in the active state. At increasing CO:O_2_ ratio (high *p*_CO_) CO wins the competitive co‐adsorption with oxygen and the surface becomes densely CO covered. On the CO‐covered surface (Figure 6d), molecular oxygen cannot dissociatively adsorb, making the reaction rate very low (inactive state). Hence, upon increasing *p*_CO_ a kinetic transition occurs from an active (oxygen covered surface) to an inactive (CO poisoned surface) steady state, and vice versa.^[^ ^185^, ^186^ ^]^ Such kinetic transitions in CO oxidation occur by propagation of reaction fronts (Figure 6c) and have been visualized by PEEM especially on single crystal surfaces^[^ ^94^, ^95^, ^96^, ^193^, ^194^, ^195^ ^]^ and, more recently, on individual grains of polycrystalline foils.^[^ ^97^, ^98^, ^179^, ^180^, ^183^, ^184^, ^185^, ^186^, ^187^, ^188^, ^189^, ^190^, ^191^, ^192^ ^]^ Below the very first PEEM study of CO oxidation on individual oxide‐supported µm‐sized Pd aggregates is discussed (Figure 13c), which reveals a new phenomenon at the metal/oxide interface.

Figures 6 and 13d–l illustrate the operando PEEM experiment. The ZrO_2_‐supported Pd agglomerates are first exposed to a constant *p*_O2_ = 1.3 × 10^−5^ mbar at *T* = 200 °C. Under these conditions, the Pd particles are oxygen‐covered (low work function), which is why they appear bright (Figure 13e). Upon increasing the CO pressure, a kinetic transition occurs from active (O‐covered) to inactive (CO‐covered; high work function) Pd, reflected by the change to dark contrast in PEEM. This is shown in Figure 13e–g for a chosen Pd aggregate. Because CO_2_ is formed only on the O‐covered and not on the CO‐covered Pd surface, the reaction rate is directly reflected in the PEEM intensity. The particular CO pressure τ_A_ (Figures 6a and 13h), at which the kinetic transition occurs, can be determined from the PEEM‐video files. The deactivation front starts at *p*_CO_ = 4 × 10^−5^ mbar (Figure 13h) on the ZrO_2_‐supported Pd aggregate, but other aggregates in the field‐of‐view behaved identically. Upon lowering the CO pressure to τ_B_ (τ_B_ < τ_A_, due to hysteresis behavior), another kinetic transition (back to the O‐covered state with bright contrast) restores high activity (cf. Figure 6a).

Interestingly, the CO‐tolerance (represented by *p*_CO_) depends on the support. When an “inactive” Pt foil was used as support (inactive as under the current conditions Pt is constantly CO‐poisoned; Figure 13i), the Pd particles behave like an unsupported rough (sputtered) Pd surface.^[^ ^192^ ^]^ Most importantly, for the “quasi‐unsupported” Pd particles on Pt, the CO‐poisoning already occurs at lower τ_A_ (*p*_CO_ = 2 × 10^−5^ mbar), indicating less CO tolerance (Figure 13j‐l). Note that at this CO pressure, the ZrO_2_ supported Pd remains active (oxygen covered) (Figure 13f). Apparently, on ZrO_2_‐supported Pd, CO‐poisoning occurs at a CO pressure that is two times higher than on quasi‐unsupported Pd (Figure 13h), demonstrating that that the oxide support makes Pd a much better CO oxidation catalyst. For Pd/Al_2_O_3_, a similar beneficial support effect was found, but with different magnitude.^[^ ^183^ ^]^ Because the Pd powder aggregates are the same in all samples, an effect of different surface morphology or roughness can be excluded.

**Figure 14:**
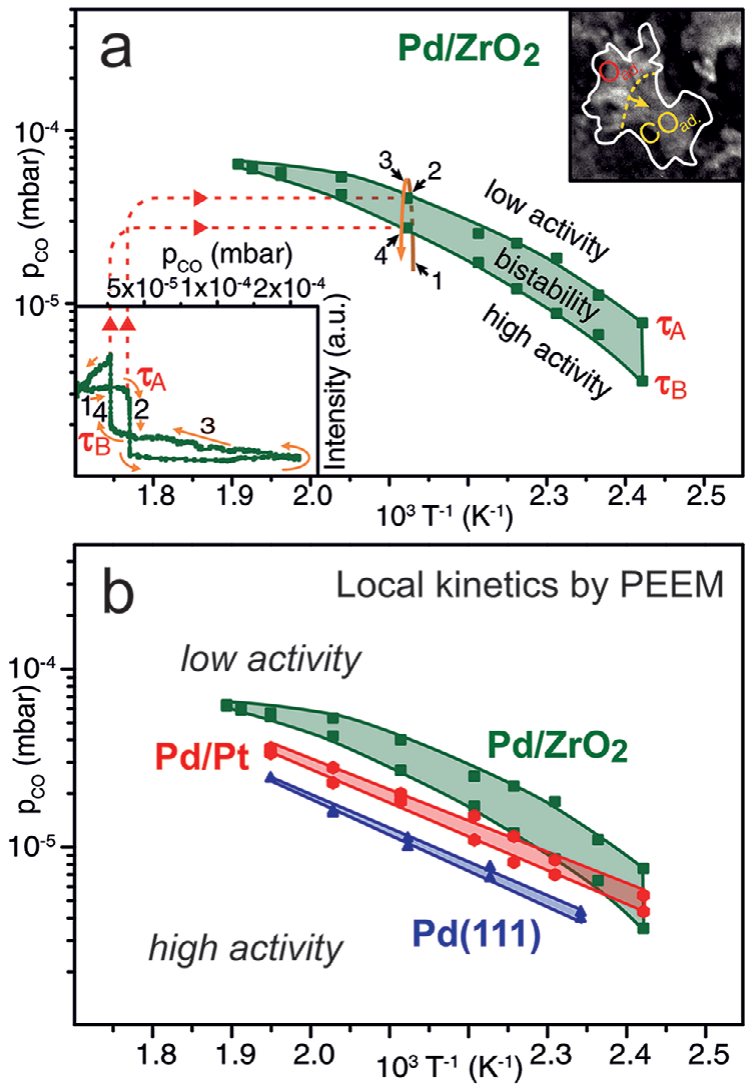
Local kinetic data for CO oxidation on Pd, derived from PEEM kinetics by imaging. a) Kinetic phase diagram for CO oxidation on the individual Pd agglomerate supported by ZrO_2_ marked in Figure 13d (*p*_O2_ = 1.3 × 10^−5^ mbar). The left inset displays how the *t*_A_ and *t*_B_ values are deduced from the PEEM intensity hysteresis during a cycle‐wise variation of *p*_CO_ at 200 °C. The right inset shows the kinetic transition *t*_B_ from the inactive to the active steady state. b) comparison of catalytic behaviour of Pd/ZrO_2_, Pd supported on Pt and a Pd(111) domain of a polycrystalline Pd foil. Adapted with permission.^[^ ^183^ ^]^ Copyright 2018, Springer Nature.

The results of analogous measurements at various temperatures on Pd/ZrO_2_, Pd/Pt and Pd(111) are summarized in **Figure** **14** as kinetic phase diagrams in the *p*_CO_ */T^−1^* parameter space, displaying the temperature‐dependent τ_A_ and τ_B_ values. The diagrams display regions of the active steady state, inactive steady state and of bistability, clearly revealing a promoting effect by the support (the higher up in the graph a region is, the more CO tolerant the catalyst is). The promotion was further confirmed by simultaneously measured global mass spectroscopic (MS) CO_2_ formation, averaged over the entire ≈10 × 10 mm^2^ catalyst surface (Figure 6a).^[^ ^183^ ^]^

**Figure 15:**
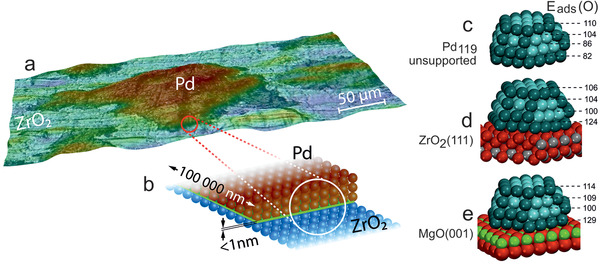
Origin of the long‐ranging effect of the nano‐scale metal/oxide interface on CO oxidation on Pd, reaching over hundreds of µm. a) optical micrograph and b) schematic illustration. Results of DFT calculations of oxygen adsorption energies on c) unsupported, d) cubic ZrO_2_(111)‐supported and e) MgO(100)‐supported Pd_119_ particles with respect to ½O_2_ gas phase molecule. The values of *E*_ads_(O) in kJ mol^−1^ are calculated for fcc sites. Pd atoms on edges and corners are displayed as turquoise spheres and those on terraces as cyan spheres; Zr, O, and Mg atoms are displayed as grey, red and green spheres, respectively. Adapted with permission.^[^ ^183^ ^]^ Copyright 2018, Springer Nature.

Now, the physical origin of the promotion at the interface needs to be evaluated. Returning to the PEEM observations, one should note that for both oxide‐supported and Pt‐supported Pd aggregates the deactivation fronts always start at the perimeter sites, which points to their special role (**Figure** **15**a). However, based on numerous reports of locally higher catalytic activity of perimeter sites, it is counterintuitive that the perimeter sites become deactivated (poisoned) first. One would rather expect that upon CO‐poisoning the perimeter remains active, whereas other (non‐interface) sites would be inactive (so that PEEM should show a bright (active) perimeter of dark (inactive) Pd particles). However, PEEM clearly discarded this scenario and directly visualized that both deactivation and reactivation fronts started at the metal/oxide perimeter sites, but then propagated over the entire 10–200 µm‐sized Pd aggregate (Figure 13d–l). In other words, the metal/oxide perimeter governed the initiation of poisoning, but then the process (reaction front) just proceeds over hundreds of micrometers.

Apparently, under τ_A_ conditions, it is easier for CO to win the co‐adsorption competition with O at the perimeter sites than on the interior of particle facets, further away from the interface. This is rationalized considering the geometrical restrictions of metal/oxide interfaces. First, on the active oxygen‐covered Pd surface, adsorbed CO molecules on the perimeter are surrounded by less O adatoms than CO adsorbed on the facet interior (i.e., by 4 oxygen atoms instead of 6), so they are oxidized less rapidly (Figure 15b). Second, dissociative adsorption of molecular oxygen requires two free adjacent Pd hollow sites, which are significantly less probable to find on the perimeter than on a terrace. It is thus easier for CO to repopulate/poison sites at the perimeter than on a terrace.

As deactivation always starts at the interface, the onset of this process must also depend on the type of metal/oxide interface. To rationalize the specific properties of the metal perimeter sites, DFT calculations of adsorption energies of CO molecules and O atoms on unsupported and oxide‐supported Pd nanoparticles were performed (Figure 15 c‐e).^[^ ^183^ ^]^ Pd aggregates were modelled as truncated cuboctahedral Pd_119_ particles (fcc structure), with a {111} interface with the oxide support. These particles are large enough to represent bulk‐like particles, but still small enough to model particles of technological catalyst.^[^ ^33^ ^]^

The computed CO adsorption energies are rather similar on most sites, whereas the adsorption energies of O atoms, E_ads_(O), on fcc three‐fold hollow sites on Pd{111} nanofacets (Figure 15c‐e) indeed vary. On the top particle facet *E*_ads_(O) is about 110 kJ mol^−1^ (irrespective of the support), which is close to *E*_ads_(O) of 98 kJ mol^−1^ on Pd(111), because the support does not affect O adsorption on sites distant from the interface. In contrast, closer to the interface, *E*_ads_(O) is about 40 kJ mol^−1^ higher on oxide supported Pd particles than on the respective sites of unsupported Pd_119_. The stronger O bonding on the perimeter sites of oxide‐supported Pd aggregates thus favors oxygen adsorption and counteracts CO poisoning. This prevents/delays the initiation of CO‐poisoning and propagation of the deactivation front, making the entire oxide‐supported Pd aggregates more CO‐tolerant than unsupported aggregates. Consequently, the effect of the perimeter sites is not locally‐restricted to the nanometer‐scale boundary, but the perimeter makes the entire µm‐sized Pd aggregates more CO tolerant than unsupported aggregates.

In this way, the local nanoscale metal/oxide interaction affects the catalytic activity of metal sites thousands of nanometers away from the interface. The communication between interface and interior surface sites is transmitted via adsorbate diffusion (propagating waves/reaction fronts). Still, it is astonishing that a minority species, with the perimeter sites comprising just < 0.001% of all Pd surface sites on the meso‐scale aggregates, improves the global CO tolerance of the entire catalyst. It is important to note, that the nanoscale interface will similarly influence the catalytic performance of particles in the nm size range. For the CO tolerance, the initiation of the CO deactivation front is critical, because after initiation the front just proceeds, independent of the facet size.

**Figure 16:**
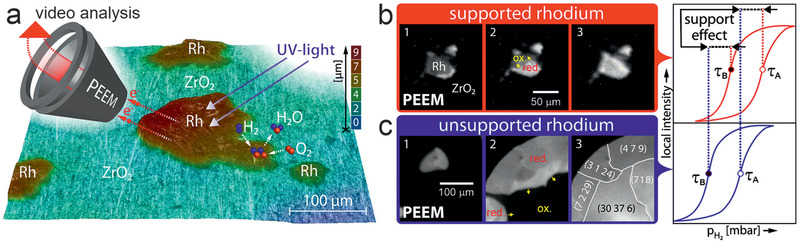
a) Illustrated optical 3D image of meso‐scale Rh particles supported by ZrO_2_. The catalytic H_2_ oxidation reaction on Rh is visualized in situ by PEEM. b) Oxide supported Rh particles: the row of video‐frames illustrates the kinetic transition τ_A_ from the inactive to the active steady state upon variation of the H_2_ pressure between 1 × 10^−7^ and 8 × 10^−6^ mbar at 180 °C and constant *p*_O2_ of 7.7 × 10^−7^ mbar. The inactive (oxygen covered) Rh surface is marked with “ox.” in frame 2, the active Rh surface with “red”. The corresponding PEEM intensity curve is shown on the right. c) Analogous measurements for unsupported sputtered polycrystalline Rh foil. Adapted with permission.^[^ ^184^ ^]^ Copyright 2018, Elsevier.

An analogous communication effect was observed for ZrO_2_ supported meso‐scale Rh particles in H_2_ oxidation (**Figure** **16**a), once more a prototypical Langmuir‐Hinshelwood reaction.^[^ ^184^ ^]^ This is evident from the PEEM images/videos of the ongoing reaction, showing that an activating H front initiates at the metal/oxide interface of the Rh particles (Figure 16b) or at defects within individual domains of a Rh foil (Figure 16c; note that on Rh H appears bright and O appears dark). However, adsorbed O acts as a poison for dissociative hydrogen adsorption, so that the stronger O bonding at metal/oxide interfaces makes the Rh particles less active than an unsupported Rh. In other words, due to the support effect, higher *p*_H2_ is required to initiate the kinetic phase transition via activating H fronts on Rh particles (≈2 × 10^−6^ mbar) than on sputtered Rh foil (≈1 × 10^−6^ mbar). Resulting from the unequal adsorption properties of oxygen and hydrogen, a cyclewise variation of *p*_H2_ (at constant *p*_O2_ and *T*) leads to a hysteresis of the reaction rate (right panels of Figure 16b,c).^[^ ^184^ ^]^ For kinetic phase diagrams see.^[^ ^184^ ^]^

In summary, a long‐range effect of metal/oxide boundaries on the reactivity of Pd/ZrO_2_ in CO oxidation and of Rh/ZrO_2_ in H_2_ oxidation was directly imaged by operando surface microscopy by PEEM. Although the initiation of CO‐poisoning fronts occurs at the perimeter sites, stronger oxygen binding at the perimeter sites of oxide‐supported Pd particles (derived from DFT) explains their higher CO‐tolerance. As long as catalyst deactivation is not initiated, metal sites on oxide‐supported Pd particles maintain high catalytic activity at increased CO pressure, even if they are as far as several tens of µm away from the metal‐oxide boundary. Thus, the higher CO tolerance of the metal/oxide perimeter sites makes entire µm‐sized Pd particles more resistant to CO poisoning. In contrast, the stronger bonding of O at the perimeter sites of oxide‐supported Rh particles “delays” the initiation of activating H reaction fronts. The key observation in both operando studies is a long‐range effect of the nanoscopic metal/oxide interface, which may help to further exploit metal‐support interactions for improving supported catalysts. Apparently, such local effects can not be addressed by area‐averaging methods, even when applied under reaction conditions.

### 4.4 Further Developments

The operando approach has reached a high level of maturity, but there is always room for improvement. Clearly, advances in sensitivity, energy‐, time‐, and spatial‐resolution are beneficial, especially as the active sites and active species often seem short‐lived minorities. In XAS, for example, utilizing higher flux of high energy photons enables better resolved detection schemes, such high energy resolution fluorescence detection (HERFD)‐XAS (instead of the conventional total fluorescence mode).^[^ ^51^, ^54^, ^132^, ^133^, ^134^, ^135^ ^]^ As schematically shown in Figure 3a, the spectrometer is equipped with a set of four bent Ge(555) analyzer crystals (with the energy resolution further improved by masks in front of each of them). Because fluorescence‐emitted rather than absorbed radiation is detected, the lifetime broadening is minimized. The overall bandwidth is ≈0.6 eV (FWHM), which is about ten times lower than that expected from the core hole lifetime. Additional features can then be resolved, for example, in studies of oxide supported Au clusters.^[^ ^175^, ^293^ ^]^

**Figure 17:**
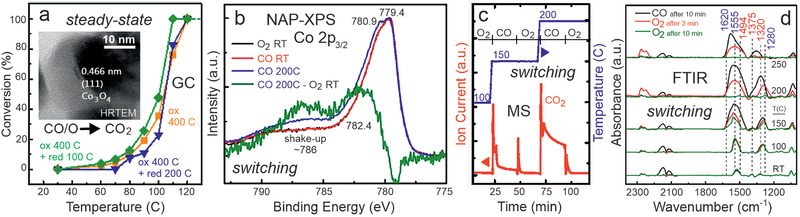
a) CO oxidation activity of Co_3_O_4_ in 5 vol.% CO, 10 vol.% O_2_ and 85 vol.% He (total flow 50 mL min^−1^) after different pretreatments. The inset shows a HRTEM image. b) Operando NAP‐XPS on Co_3_O_4_ during O_2_/CO switching (0.15 mbar O_2_ versus 0.15 mbar CO; hν = 1015 eV; KE = 200 eV; probing depth ≈0.6 nm): comparison in O_2_ and CO at RT and 200 °C, and difference spectrum (CO 200 °C‐ O_2_ RT). c) Corresponding catalytic MS data: evolution of CO_2_ upon switching between O_2_ and CO at different temperatures. d) Operando FTIR on Co_3_O_4_ upon CO/O_2_ switching (50 mbar CO vs 50 mbar O_2_): spectra recorded during the 10th minute of CO exposure (black), during the 2nd minute of O_2_ exposure (red) and during the 10th minute of O_2_ exposure (green). Monodentates in red, bidentates in blue. Adapted with permission.^[^ ^59^ ^]^ Copyright 2018, American Chemical Society.

In the case studies of operando spectroscopy discussed so far, mainly steady‐state XAS, IR, and NAP‐XPS spectra were presented (typically at various temperature), with acquisition times on the order of minutes. This also holds true for a study of CO oxidation on a Co_3_O_4_ spinel catalyst with an average crystallite size of 28 nm (HRTEM inset in **Figure** **17**a).^[^ ^59^, ^294^ ^]^ Activity measurements by GC indicated that the catalyst was highly active already around 100 °C, with a minor effect of oxidative versus oxidative/reductive pretreatment (Figure 17a). Operando steady‐state NAP‐XPS and IR spectra (not shown) solely indicated a fully oxidized spinel surface and the presence of mainly monodentate carbonates, but without significant changes around the ignition temperature. In preceeding studies of various cobalt oxide catalysts, the nature of the active sites has been debated, such as the role of Co^3+^ versus Co^2+^ and of oxygen vacancies, and especially their surface concentrations under specific reaction conditions. Furthermore, size and shape effects were reported.^[^ ^59^, ^131^, ^294^, ^295^, ^296^ ^]^

However, additional insight can be gained by *switching* between O_2_ and CO atmosphere, simultaneously recording NAP‐XPS Co 2p spectra (kinetic energy (KE) = 200 eV; Figure 17b) and MS of CO_2_ (Figure 17c). Still, the spectral changes were rather subtle. When switching from O_2_ to CO at 200 °C, the Co 2p_3/2_ peak slightly shifted to higher BE, indicating a 15% decrease of Co^3+^ at/near the surface (peak assignment:^[^ ^297^ ^]^ Co^3+^ 779.4 eV, Co^2+^ 780.9 eV, Co^2+^ in Co(OH)_2_ 782.4 eV, shake‐up characteristic of Co^2+^ ≈786 eV). To illustrate how small the maximum change in surface oxidation state is, Figure 17b compares NAP‐XPS spectra in pure CO at 200 °C and in pure O_2_ at RT (after oxidative pretreatment at 400 °C). The difference spectrum shows the presence of features related to increased Co^2+^ (782.4 eV and shake‐up at ≈786 eV). This indicates that CO reduces part of the surface, while O_2_ reoxidizes it, as expected for a MvK mechanism.^[^ ^295^, ^296^ ^]^ Nevertheless, the active sites were apparently *minority species* even at 200 °C, given that full conversion was already reached at ≈110 °C.

Simultaneously recorded MS data support this picture (Figure 17c): upon switching from O_2_ to CO, a sharp CO_2_ peak followed by a slow decrease points to fast reaction of CO with rapidly consumed surface oxygen and a slower continuous reaction with bulk oxygen diffusing to the surface. In the opposite case, when O_2_ was dosed after previous CO exposure, there was only minor CO_2_ production due to surface carbonate decomposition. The role of the adsorbed carbonates was examined analogously by operando FTIR from RT to 250 °C (Figure 17d), by CO/O_2_ switching (50 mbar CO versus 50 mbar O_2_).^[^ ^59^ ^]^ The mainly monodentate surface carbonates were rather stable spectators, only disappearing at temperatures way above 100 °C. Interestingly, the carbonates only formed under reaction conditions or by CO exposure, but not via the re‐adsorption of the product CO_2_. A discussion of possible reaction pathways can be found in the literature.^[^ ^59^ ^]^

**Figure 18:**
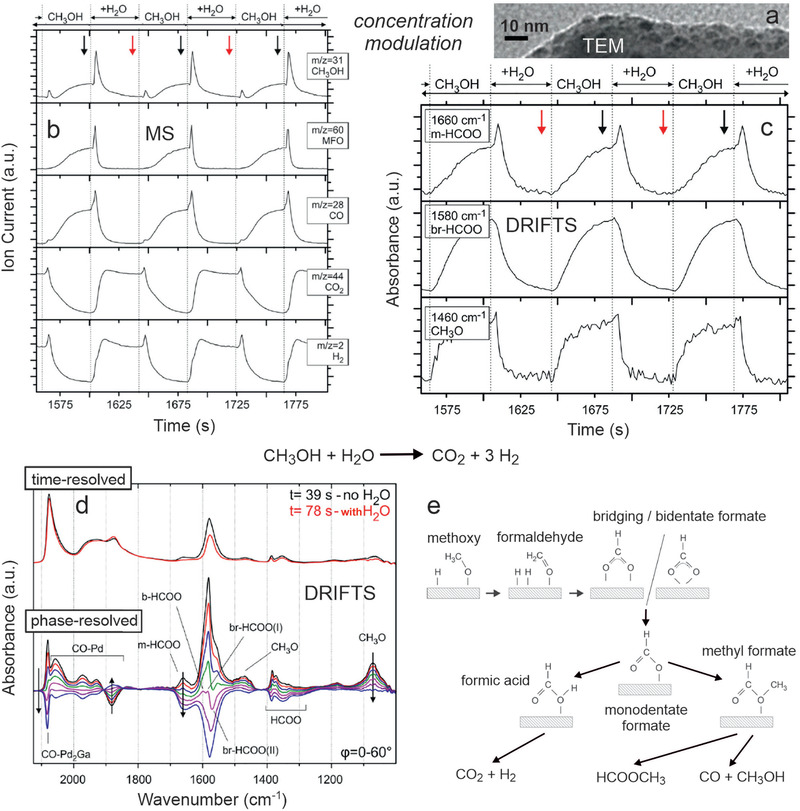
a) TEM Micrograph of Pd_2_Ga/Ga_2_O_3_. b) MS and c) corresponding DRIFTS traces recorded during three modulation periods (82 s each) of pulsing H_2_O/Ar into a steady CH_3_OH/Ar flow (1%CH_3_OH/Ar vs 1%CH_3_OH/1%H_2_O/Ar at 250 °C). Arrows indicate when the spectra used to calculate the averaged spectra of d) (top) were recorded. d) Two selected averaged time‐resolved spectra recorded at the end of each half‐period (top) and corresponding phase‐resolved spectra in a phase angle range of 0–60° calculated using phase sensitive detection (PSD). e) Scheme of mechanistic insights from operando concentration modulation IR. Adapted with permission.^[^ ^72^ ^]^ Copyright 2012, American Chemical Society.

In a similar way, selective methanol steam reforming (MSR) to CO_2_/H_2_ on Pd_2_Ga/Ga_2_O_3_ catalysts was examined (**Figure** **18**).^[^ ^60^, ^72^ ^]^ The intermetallic Pd_2_Ga nanoparticles (Figure 18a; size ≈3 nm) were formed by reducing 2 wt% Pd/Ga_2_O_3_ in hydrogen at 400 °C (supported Pd just produces undesired CO/H_2_ and methylformate (MFO) in MSR).^[^ ^60^ ^]^ Upon switching from CH_3_OH/H_2_O/He to He at 225 °C, transmission FTIR indicated that adsorbed methoxy and monodentate (m)‐formate decreased rapidly within 2 minutes, pointing to their active involvement in the reaction mechanism, whereas bridging (br)‐ and bidentate (b)‐formate decreased very slowly (not shown, see ref. ^[^ ^72^ ^]^). Still, it is difficult to judge on the exact role of these species.

When ongoing technological progress allowed to reduce the spectra acquisition time to seconds or below, adsorbate changes induced by rapid alterations in gas feed or temperature could be observed in real time. With respect to time‐resolution and the observation of dynamic (transient) changes, a prime methodology is modulation excitation spectroscopy (MES), also called demodulation.^[^ ^298^, ^299^ ^]^ A process variable is periodically alternated and the correlation between the catalytic performance and the acquired spectra is evaluated. This enables to differentiate active species (periodically responding to the modulation) and spectator species (not responding to the modulation). Below, the feed gas composition is modulated by computer‐controlled solenoid valves, electronically triggered via the IR spectrometer´s rapid scan option. Similarly, temperature or pressure can be modulated and MES has been successfully applied to ATR‐IR, DRIFTS, Raman, XRD, or XAS.^[^ ^167^, ^168^, ^300^, ^301^, ^302^ ^]^

An example of concentration‐modulation spectroscopy is shown in Figure 18, which enabled to refine the insight on MSR on Pd_2_Ga/Ga_2_O_3_. Figure 18b displays MS traces recorded during modulating the feed between CH_3_OH/Ar and CH_3_OH/H_2_O/Ar at 250 °C. Upon switching to MSR conditions, the spikes in methanol, methyl formate (MFO) and CO indicate that during CH_3_OH exposure a species accumulates that then rapidly reacts upon water addition (suggesting HCOOCH_3_ → CH_3_OH + CO to occur). Mainly thereafter, CO_2_/H_2_ are produced, which decreased when the feed was changed back to CH_3_OH/Ar. Simultaneously, in each modulation period of 82 s, 60 consecutive DRIFTS spectra were acquired. The vibrational bands characterising methoxy, monodentate and bridging formate revealed their active involvement in the reaction mechanism (Figure 18c). In the presence of water, methoxy and bridging formate simply decreased, but monodentate formate “spiked”, indicating fast conversion of bridging to monodentate formate by water. Note that the MS spikes in the formation of MFO, CH_3_OH, and CO (Figure 18b) are paralleled by the IR spike of monodentate formate (Figure 18c). The reaction sequence becomes clearer from Figure 18d, contrasting time‐resolved and phase‐resolved detection. Figure 18d (top) displays two selected averaged time‐resolved spectra, recorded at the end of each half period. The arrows in Figure 18c indicate when the IR spectra used to calculate the averaged spectra were taken (averaging over a number of modulation periods apparently increases the signal to noise ratio). All measured and averaged time‐resolved spectra were then mathematically converted into phase‐resolved spectra using phase‐sensitive detection (PSD),^[^ ^298^, ^299^ ^]^ as shown in Figure 18d (bottom).

PSD allows to identify active species responding to the external stimulation, in the current case the modulation of the H_2_O feed concentration, whereas spectators exhibit no response to the modulation (i.e., static signals do not appear in phase‐resolved spectra). The relevant surface species can be further differentiated by the phase delay between excitation and their spectral response. A comparison of time‐ and phase‐resolved spectra in Figure 18d reveals that carbonyls exhibit the strongest signals in the time‐resolved spectra, but that formates and methoxy are much more intense in the phase‐resolved ones (i.e., they more strongly respond to the H_2_O modulation and are more likely involved in the catalytic cycle). In addition, features are resolved that are invisible in time‐resolved mode, as both the carbonyl and formate regions show much more detail in the PSD spectra. The specific roles of Pd_2_Ga and Ga_2_O_3_ still hold open questions, but the high temperature H_2_ pretreatment that forms the intermetallics (by partial support reduction around the Pd nanoparticles and GaO*_x_* reacting with Pd^[^ ^60^ ^]^) seems crucial for creating the active sites on Ga_2_O_3_, strongly enhancing the formation of formates during MSR.^[^ ^72^ ^]^ Furthermore, on Pd_2_Ga, the reaction to CO/MFO is limited.

Altogether this allowed to suggest the reaction mechanism displayed in Figure 18e. Adsorbed CH_3_O, likely via formaldehyde, reacts with either lattice O of Ga_2_O_3_ or surface OH groups to stable bridging‐/bidentate formates, which upon H_2_O addition are converted to monodentate species. These react with adsorbed CH_3_O to methyl formate or with OH groups (from H_2_O) to CO_2_ and H_2_ (the intermediate formic acid was not detected, though). The preferred selective reaction with OH is faster, leading to a smaller concentration of m‐HCOO under MSR conditions (i.e., in the presence of H_2_O). Adsorbed MFO can desorb or decompose to methanol and CO and is thus not an intermediate in selective MSR (also carbonyls play a minor role in the selective reaction). Clearly, such insight can not be obtained from pre‐ or post‐reaction (co‐)adsorption studies, once more demonstrating the importance of the operando concept.

Apparently, shorter acquisition times are also beneficial for XAS and using a faster monochromator allows to record operando quick scanning “QEXAFS”.^[^ ^54^, ^55^ ^]^ In a study of MSR on Pd/ZnO,^[^ ^20^, ^117^ ^]^ operando QEXAFS was employed to monitor the formation of the active intermetallic PdZn phase in real time, paralleled by a selectivity change from unwanted CO/H_2_ to desired CO_2_/H_2_. It was also directly proven that oxygen exposure, often used for catalyst cleaning/activation, in fact decomposed the selective PdZn phase into unselective palladium metal and ZnO. However, the PdZn intermetallic re‐formed during continued MSR reaction. In situ QEXAFS studies of Pt/CeO_2_/Al_2_O_3_ revealed that the metal/oxide interface facilitated ceria reduction.^[^ ^303^ ^]^ Q‐XAS and operando FTIR were employed to explain the effect of Ni nanoparticle size in CO_2_ hydrogenation.^[^ ^82e^ ^]^ MES and QEXAFS were even combined to gain unprecedented insight in methane partial oxidation catalysts (CoMoS, NiMoS).^[^ ^302^ ^]^

To this point, several operando methods were combined in one study, that is, XAS, IR, or NAP‐XPS were applied to the same/analogous sample one after the other (each in combination with MS or GC, of course). Clearly, the simultaneous application of two operando methods is specifically powerful. For example, combined XAS/DRIFTS,^[^ ^168^, ^303^, ^304^, ^305^ ^]^ XAS/XRD,^[^ ^135^ ^]^ XRD/Raman,^[^ ^56^ ^]^ or EPR/UV–vis/Raman^[^ ^81^ ^]^ (or even more/other combinations)^[^ ^76^, ^306^ ^]^ enable correlating various pieces of information on atomic and electronic structure, composition, and adsorbates.

Finally turning to improved spatial resolution, employing focused X‐ray beams enables microspectroscopy, so that several locations within a sample can be analyzed.^[^ ^51^, ^85^ ^]^ This is specifically important when the conversion and thus the feed gas composition change along the catalyst bed, which may affect catalyst composition. Even more, X‐ray microscopy on the meso‐ to macro‐scale can be provided by high brilliance synchrotron radiation sources.^[^ ^85c^ ^]^ Operando electron microscopy can be performed in scanning (ESEM) or transmission (OTEM), the latter utilizing dedicated microreactors with electron transparent windows, coupled to sensitive MS analysis.^[^ ^91^, ^92^, ^93^, ^271^, ^272^ ^]^ NAP‐PEEM is still a niché, but will find more applications for sure.^[^ ^197^, ^198^ ^]^ Altogether, the ongoing technological development of new and more sensitive operando methods, both in time‐ and spatially‐resolved modes, will enable new insights on active centers in catalytic systems. This is a prerequisite of catalysts improvement and finally rational design.

## 5 Synopsis

Despite the seemingly simple catalyst composition, the discussed case studies of Au/CeO_2_, CuNi/ZrO_2_, Pd/ZrO_2_, Rh/ZrO_2_, Co_3_O_4_, and Pd_2_Ga/Ga_2_O_3_ exhibit a multitude of complexities along various directions. These include atomic structure, surface composition, vacancies and metal/oxide interfaces, the exact details of which depend on synthesis, pretreatment (activation), and reaction conditions (poisoning/deactivation). Clearly, only operando (averaging) surface spectroscopy (XAS, NAP‐XPS, IR) and (spatially‐resolved) surface microscopy (PEEM), per definitionem combined with simultaneous reactant/product analysis (MS, GC), can provide direct and meaningful insight into the relevant catalyst working state. Correlating information on the catalyst structure/composition/adsorbed species and the catalytic performance in steady‐state provide hints on the reaction mechanism, that can then be further evaluated by detailed kinetic analysis. Concentration modulation spectroscopy is a powerful tool to differentiate active species from spectators. The simultaneous application of two operando methods in a single experiment currently gains more and more momentum. Theory (DFT) is indispensable for the analysis of the operando data and for critically assessing experiments, in the best case enabling to verify (steps of) a reaction mechanism.

The discussed examples were deliberately selected to bridge a wide range of length‐scales: Au_38_ clusters versus CuNi/Pd_2_Ga/Co_3_O_4_ nanoparticles (with several thousand atoms) versus µm‐sized Pd/Rh powder aggregates (with quadrillions of atoms). For Au_38_/CeO_2_, activity in CO oxidation was controlled by ligand decomposition and atom (S, Au) migration to the support during oxidative activation and reaction. For CuNi/ZrO_2_, with Cu decoration intended to limit coking, Ni surface segregation occurs during methane decomposition, which must be accounted for in the synthesis. Still, there is a trade‐off between activity and coking resistance. On Co_3_O_4_, the active sites of CO oxidation seem to be minority species, involving the Co^3+^/Co^2+^ redox couple and oxygen vacancy formation, with carbonates acting solely as spectators. For Pd_2_Ga/Ga_2_O_3,_ a selective MSR reaction sequence was identified. For Pd/ZrO_2_, the metal/oxide interface steers the CO‐tolerance of an entire µm‐sized particle, explaining why an active interface is so important. Vice versa, for hydrogen oxidation on Rh/ZrO_2_, the stronger bonding of interfacial O rather hinders reactivity. Clearly, the perimeter sites and the facets of a particle are coupled by diffusion (reaction fronts) and cannot be considered as separate entities.

Both for operando surface spectroscopy and surface microscopy one strives for even higher energy‐, time‐, and spatial‐resolution, typically requiring synchrotron radiation. Combining different methods within one measurement, resonantly enhanced modes, modulation‐excitation, and demanding environments (high gas pressures, temperatures, liquid phase, light illumination, etc.) are technically challenging, but will have enormous gain and impact. The advances in operando surface microscopy truly enabled a transition from “imagining to imaging a working surface”.^[^ ^307^ ^]^

## Conflict of Interest

The author declares no conflict of interest.
